# Leitlinie S1: Long COVID: Differenzialdiagnostik und Behandlungsstrategien

**DOI:** 10.1007/s00508-021-01974-0

**Published:** 2021-12-01

**Authors:** Susanne Rabady, Johann Altenberger, Markus Brose, Doris-Maria Denk-Linnert, Elisabeth Fertl, Florian Götzinger, Maria de la Cruz Gomez Pellin, Benedikt Hofbaur, Kathryn Hoffmann, Renate Hoffmann-Dorninger, Rembert Koczulla, Oliver Lammel, Bernd Lamprecht, Judith Löffler-Ragg, Christian A. Müller, Stefanie Poggenburg, Hans Rittmannsberger, Paul Sator, Volker Strenger, Karin Vonbank, Johannes Wancata, Thomas Weber, Jörg Weber, Günter Weiss, Maria Wendler, Ralf-Harun Zwick

**Affiliations:** 1 Department Allgemeine Gesundheitsstudien, Kompetenzzentrum für Allgemein- und Familienmedizin, Karl Landsteiner Privatuniversität für Gesundheitswissenschaften, Dr. Karl-Dorrek-Straße 30, 3500 Krems, Österreich; 2 Pensionsversicherungsanstalt, Rehabilitationszentrum Großgmain, Großgmain, Österreich; 3 Klinische Abteilung Phoniatrie-Logopädie, Universitätsklinik für Hals‑, Nasen- und Ohrenkrankheiten, Medizinische Universität Wien, Wien, Österreich; 4 Neurologische Abteilung, Klinik Landstraße, Wiener Gesundheitsverbund, Wien, Österreich; 5 Abteilung für Kinder- und Jugendheilkunde, Klinik Ottakring, Wiener Gesundheitsverbund, Wien, Österreich; 6 Unit Versorgungsforschung in der Primärversorgung, Zentrum für Public Health, Medizinische Universität Wien, Wien, Österreich; 7 Praxis Dr. Hofbaur, Arbesbach, Österreich; 8 Unit Health Services Research and Telemedicine in Primary Care, Department of Preventive- and Social Medicine, Center for Public Health, Medical University of Vienna, Wien, Österreich; 9 Praxis Dr. Hoffmann-Dorninger, Wien, Österreich; 10 Abteilung für Pneumologische Rehabilitation, Philipps Universität Marburg, Marburg, Deutschland; 11 Praxis Dr Oliver Lammel, Ramsau am Dachstein, Österreich; 12 Klinik für Lungenheilkunde, Kepler Universitätsklinikum, Linz, Österreich; 13 Universitätsklinik für Innere Medizin II, Innsbruck, Österreich; 14 Universitätsklinik für Hals‑, Nasen- und Ohrenkrankheiten, Medizinische Universität Wien, Wien, Österreich; 15 Ordination Dr. Stephanie Poggenburg, Hart bei Graz, Österreich; 16 Abteilung Psychiatrie und Psychotherapie, Pyhrn-Eisenwurzen-Klinikum, Steyr, Österreich; 17 Dermatologische Abteilung, Klinik Hietzing, Wiener Gesundheitsverbund, Wien, Österreich; 18 Universitätsklinik für Kinder- und Jugendheilkunde, Medizinische Universität Graz, Graz, Österreich; 19 Klinische Abteilung für Pulmologie, Universitätsklinik für Innere Medizin II, Medizinische Universität Wien, Wien, Österreich; 20 Klinische Abteilung für Sozialpsychiatrie, Medizinische Universität Wien, Wien, Österreich; 21 Abteilung für Innere Medizin 2 (Kardiologie, Intensivmedizin), Klinikum Wels-Grieskirchen, Wels, Österreich; 22 Klinikum Klagenfurt, Feschnigstraße 11, 9020 Klagenfurt, Österreich; 23 Univ.-Klinik für Innere Medizin II, Medizinische Universität Innsbruck, Innsbruck, Österreich; 24 Ambulante internistische Rehabilitation, Therme Wien Med, Wien, Österreich

**Keywords:** Long COVID, Primary care, Diagnostics, Treatment, Rehabilitation, COVID-sequelae, Post infection syndrome, Long COVID, Primary care, Diagnostik, Behandlung, Rehabilitation, COVID-Folgen, Postinfektiöses Syndrom

## Abstract

Die vorliegende Leitlinie S1 fasst den Stand der Kenntnis zu Long COVID zum Zeitpunkt des Redaktionsschlusses zusammen. Aufgund der starken Dynamik der Wissensentwicklung versteht sie sich als „living guideline“. Der Schwerpunkt liegt auf der praktischen Anwendbarkeit auf der Ebene der hausärztlichen Primärversorgung, die als geeignete Stelle für den Erstzutritt und für die primäre Betreuung und Behandlung verstanden wird. Die Leitlinie gibt Empfehlungen zur Differenzialdiagnostik der häufigsten Symptome, die in der Folge einer Infektion mit SARS-CoV‑2 auftreten können, zu therapeutischen Optionen, zu Patient:innenführung und -betreuung, sowie zu Wiedereingliederung in den Alltag, und die Rehabilitation. Entsprechend des Krankheitsbildes ist die Leitlinie in einem interdisziplinären Prozess entstanden und gibt Empfehlungen zu Schnittstellen und Kooperationsmöglichkeiten.

## Abstract (en)

This guideline comprises the state of science at the time of the editorial deadline. In view of the high turnover of knowledge the guideline is designed as a living guideline. The main objective was to provide a tool for the use in primary care, being considered well suited as a first point of entry and for the provision of care. The guideline gives recommendations on the differential diagnosis of symptoms following SARS-CoV‑2 infection, on their therapeutic options, as well as for guidance and care of the patients concerned. It also offers advice concerning return to daily life and rehabilitation. Long COVID being a very variable condition, we chose an interdisciplinary approach.

## 1. Einführung

Viele Patient:innen benötigen lange Zeit für die Genesung nach COVID‑19. Die Symptome reichen von einer geringfügigen Leistungsminderung bis zu höhergradigen Einschränkungen sowie persistierenden Krankheitsymptomen. Die Symptome können nach derzeitiger Kenntnis sowohl nach schweren als auch nach milden und moderaten Verläufen auftreten. Sie bestehen über einige Wochen bis viele Monate. Die Beschwerden können persistierend sein, rezidivierend, undulierend, oder neu aufgetreten [1–3].

**Diese Leitlinie befasst sich mit Long COVID nach milden bis moderaten Verläufen (inkl. hospitalisierten Patient:innen), jedoch nicht mit Folgeschäden und Erkrankungen nach intensivmedizinischer Behandlung**.

### Beteiligte Fachgesellschaften

Österreichische Gesellschaft für Allgemein- und Familienmedizin (ÖGAM) (federführend)

Österreichische Gesellschaft für Pneumologie (ÖGP)

Österreichische kardiologische Gesellschaft (ÖKG)

Österreichische Gesellschaft für Kinder- und Jugendheilkunde (ÖGKJ)

Österreichische Gesellschaft für Neurologie (ÖGN)

Österreichische Gesellschaft für Hals-Nasen-Ohrenheilkunde, Kopf- und Halschirurgie (ÖGHNO)

Österreichische Gesellschaft für Psychiatrie, Psychotherapie und Psychosomatik (ÖGPP)

Österreichische Gesellschaft für Infektionskrankheiten und Tropenmedizin (OEGIT)

Bei Long-COVID handelt sich um ein multifaktorielles Krankheitsgeschehen, das nach Identifikation, Behandlung und kontinuierlicher Betreuung durch Generalist:innen verlangt – sinnvollerweise in hausärztlicher Funktion – und mitunter auch einer intensiven Einbindung von und Kooperation mit den Spezialist:innen der relevanten Fachgebiete bedarf. Multiprofessionelles und multidisziplinäres Zusammenwirken entsprechend einem individualisierten Behandlungsplan sind essenziell.

Eine Vereinheitlichung der Terminologie bzw. eine Klassifizierung ist bisher noch nicht erreicht. In vielen Publikationen werden unterschiedliche Folgen von COVID‑19 unter dem Begriff „Long COVID“ gefasst [4]: Dazu zählen Folgen schwerer Akuterkrankung und deren Komplikationen, Verschlechterung vorbestehender Grundkrankheiten, fortbestehende Symptome der Erkrankung selbst, bzw. nicht zuordenbare Folgebeschwerden aus nicht vollständig geklärten Pathomechanismen, neu aufgetretene Erkrankungen [5–7]. Andere schränken den Begriff stärker auf diejenigen Symptome ein, die klinisch dem Krankheitsbild bei COVID‑19 zuordenbar [8] und nicht organisch-strukturelle Folge schwerer Erkrankung [9] sind.

In letzter Zeit wurden mehrere Vorschläge zur Klassifizierung der Symptomatologie publiziert (zusammengefasst und laufend aktualisiert im Evidence Review des NIHR – s. Kap. 4) [2].

Klinisch ist die Differenzierung der präsentierten Symptome zwischen strukturellen Spätfolgen und Symptomen im Rahmen des Long COVID-Syndroms ebenfalls nicht einfach bzw. eine Koinzidenz möglich. Wesentlich zur Beurteilung der Bedeutung ist, dass in Studien, die Symptomverläufe untersucht haben, eine deutliche spontane Abnahme der Symptomatik im Laufe der Zeit beobachtet wird [10].

## 2. Zielsetzung

- Empfehlungen für die Abklärung und Zuordnung von Symptomen bzw. Erkrankungen in zeitlichem Zusammenhang mit einer Infektion mit SARS-CoV-2:
  - Ausschluss/Abklärung von Erkrankungen aus anderer Ursache
  - Erkennen organisch-struktureller Ursachen als Folge der Erkrankung und/oder ihrer Komplikationen
  - Erkennen einer Verschlechterung vorbestehender Grundkrankheiten im Gefolge von COVID‑19
  - Abgrenzung anhaltender unspezifischer und funktioneller Störungen nach Akuterkrankung an SARS-CoV‑2 von organisch-struktureller Ursachen
- Empfehlungen zur Behandlung der zugeordneten Störungen und Beschwerden
- Empfehlungen zu Betreuung und Coping
- Empfehlungen zur Vermeidung iatrogener Verstärkung, sowie sekundärer Chronifizierung
- Empfehlungen zu Rehabilitationsbedarf und -optionen

## 3. Aufbau

- Grundlagenwissen „Long COVID“
  - Definition und Bedeutung, Charakteristika
  - Pathomechanismen
  - Organsysteme: Auswirkungen von COVID‑19 und organspezifische Folgen
- Differenzialdiagnostik häufiger Symptome
- Behandlung, Begleitung, Betreuung
- Nachsorge/Rehabilitation

## 4. Definition „Long COVID“

Es handelt sich um ein noch junges Krankheits- bzw. Beschwerdebild, dessen Einordnung sich in einem dynamischen Stadium befindet.

„Long COVID“ wird als Synonym für das Vorhandensein von Symptomen über 4 Wochen nach Erkrankungsbeginn hinaus verwendet. Die folgende Terminologie findet derzeit häufig Verwendung [8] – sie orientiert dabei sich am zeitlichen Verlauf:

- Akuterkrankung **COVID‑19**: Befunde und Symptome von COVID‑19 bis zu 4 Wochen
- Anhaltende** Symptome von COVID‑19**: 4–12 Wochen
- **Post-COVID Syndrom**: Befunde und Symptome, die während oder nach einer Infektion mit SARS-Cov‑2 entstehen und zu den bei COVID‑19 beobachteten Symptomen passen, mehr als 12 Wochen bestehen und bei denen keine andere erkennbare Ursache vorliegt

Alternativ wird vorgeschlagen [11]:

- Akutes Post-COVID: 3–12 Wochen nach COVID‑19: Symptome über die ersten 3 Wochen nach Erkrankungsbeginn hinaus
- Chronisches Post-COVID: > 12 Wochen nach Beginn der Akuterkrankung

Weitere in der Literatur verwendete Begriffe sind z. B. „post-acute sequelae of COVID‑19“ (PASC), „Chronic COVID Syndrome“ (CCS) oder „COVID‑19 long-hauler“.

Beschrieben ist eine Vielzahl von Symptomen aus einer Reihe von Organsystemen, sowohl organischer als auch funktioneller Natur. Über die Pathophysiologie funktioneller Störungen ist wenig bekannt, auch hier werden unterschiedliche Mechanismen diskutiert (s. Kap. 7).

## 5. Bedeutung

Eine Quantifizierung des Problems ist derzeit kaum möglich – die Angaben für die Häufigkeit von Long COVID in der derzeit verfügbaren Literatur schwanken zwischen 2,3 % [10] und 89 % [12].

**Figure 1:**
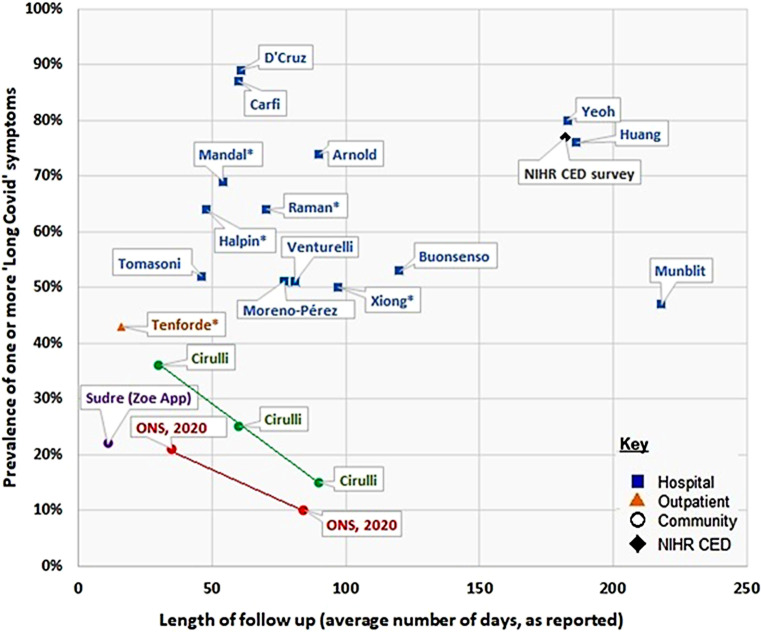

Die meisten Studien haben ihre Daten an unterschiedlichen Kollektiven erhoben (zuvor hospitalisierte Personen, nicht-hospitalisierte und gemischte Samples) und zu sehr unterschiedlichen Zeitpunkten (zwischen 3 Wochen und 9 Monaten – s. Abb. 1). Die Datenerfassung erstreckte sich vielfach über lange Zeiträume [2, 11], die Methodik der Erhebung (App, Health Records, elektronische Surveys, Interviews) ist ebenfalls so unterschiedlich, dass Vergleiche kaum möglich und Verzerrungen häufig sind.

Eine wesentliche Problematik ist die terminologische Unschärfe. Zwischen organischen Folgen schwerer Erkrankung inkl. Post Intensive Care-Syndrom, und funktionellen Störungen auch als Folge milder und moderater Verläufe, wie sie auch als Folge anderer Infektionserkrankungen (z. B. EBV, CMV) bekannt sind wird selten eine klare Trennung vollzogen, trotz der völlig unterschiedlichen Ätiologie.

Einen Überblick über die Studienlage gibt die Grafik in Abb. 1.

Noch ist nicht genau bekannt, welche Personen ein erhöhtes Risiko haben, anhaltende Symptome, die unter dem Begriff Long COVID zusammengefasst sind, zu entwickeln. Unter anderen werden auch genetische Faktoren diskutiert [5]. Für zuvor hospitalisierte und schwer erkrankte Personen dürfte die Wahrscheinlichkeit höher sein [13], was wenig überraschend erscheint, da Organschäden vornehmlich Folge schwerer Verläufe sind. Von manchen Autoren werden als weitere Risikofaktoren für einen prolongierten Krankheitsverlauf angenommen [10]:

- Symptomzahl während der akuten Erkrankungsphase [10, 14, 15]
- Alter [10, 13]
- Ausgeprägte Fatigue während der Akuterkrankung [14, 16]

Andere Autoren fanden keine Prädiktoren [6]. Auch hier dürfte die hohe Varianz bei den untersuchten Kohorten und Zeiträumen eine wesentliche Rolle spielen. Unterschiedliche prozentuelle Anteile an hospitalisierten Patient:innen in den Samples führen zu Verzerrungen, weil sich Risikofaktoren für schwere Verläufe (wie Alter, Symptomzahl, etc.) in unterschiedlichem Maß in den Ergebnissen niederschlagen.

Organische und auch funktionelle Störungen im Gefolge von Infektionskrankheiten sind keine für SARS-CoV‑2 spezifische Phänomene. Die Bedeutung von Long COVID für Gesellschaft und Gesundheitssystem liegt vor allem darin, dass während der Pandemie eine große Zahl von Personen gleichzeitig erkrankt. Bis Anfang August 2021 haben rund 650.000 Menschen in Österreich die Infektion durchgemacht. Zieht man von dieser Zahl eine geschätzte Quote von etwa 20 % [17] permanent asymptomatischer Infizierter ab und rechnet mit rund 10 % Prävalenz von Long COVID nach Wochen – also mit einem der niedrigsten Schätzwerte – so sind es mehr als 55.000 Personen in Österreich, die an *anhaltenden Symptomen unterschiedlicher Schweregrade* leiden oder gelitten haben.

In den allermeisten Fällen ist vor allem bei den funktionellen Störungen mit Spontanheilungen nach einigen Wochen bis Monate zu rechnen. Cirulli berichtet persistierende Symptome bei 36,1 % der Betroffenen nach 4 Wochen, aber nur mehr 14,6 % nach 12 Wochen [18]. Sudre findet einen ähnlichen Verlauf, findet aber insgesamt niedrigere Zahlen [10]. Andere Autoren nennen keine exakten Zahlen, beschreiben aber ähnliche Verläufe [9, 11].

Trotz der meist benignen Natur handelt es sich um ein gesellschaftlich relevantes Problem. Zur Arbeitsunfähigkeit ließ sich bisher nur eine Studie finden, welche bei hospitalisierten Personen mit COVID‑19 nach 48 Tagen eine Quote von 15 % Arbeitsunfähigkeit fand [19].

## 6. Symptomatik

Die Symptomatik, der unter dem Begriff Long COVID zusammengefassten Erscheinungen, ist inter- und intraindividuell sehr variabel, der Schweregrad reicht von Störungen des Befindens bis zu massiver Einschränkung des alltäglichen Lebens. Ein Teil der Patient:innen erholt sich nach der Erkrankung über Wochen bis Monate nicht, oder erfährt Rückfälle. Möglich sind persistierende Beschwerden ebenso wie eine undulierende Symptomatik (bekannt als „Corona-Coaster“). Bekannt ist auch ein Wieder- oder Neuauftreten von Symptomen nach einem beschwerdefreien Intervall.

Eine Reihe relevanter struktureller Erkrankungen sowie plötzliche Todesfälle nach COVID‑19 sind beschrieben [9].

Entsprechend der diskutierten Pathomechanismen der Erkrankung sind auch deren mögliche Späterscheinungen sehr vielfältig und können ganz unterschiedliche Organsysteme betreffen.

Symptome und deren Häufigkeiten werden in der Literatur ebenfalls äußerst unterschiedlich angegeben und wurden in sehr unterschiedlichen Populationen untersucht.

Weitgehende Einigkeit besteht dahingehend, dass sich der Großteil der Betroffenen nach einigen Wochen bis Monaten vollständig erholt [10, 11, 18].

Als häufigste Symptome finden sich in den meisten Studien die Folgenden [11]:

- Müdigkeit, eingeschränkte Leistungsfähigkeit, Schwäche (unterschiedlicher Schweregrade, bis zu Fatigue-Syndrom): in den meisten Studien führend [11]
- Anhaltender Verlust des Riech- und/oder Schmeckvermögens
- Atemnot (frühe Phase)
- Insomnie (späte Phase) [11]

Seltener:

- Brustschmerzen oder Brustenge
- Husten
- Arthralgien
- Muskelschmerzen
- Neuropathische Schmerzen bzw. und andere Sensibilitätsstörungen (u. a. Missempfindungen, Taubheit), beschrieben auch als „pins and needles and numbness“

Weitere Symptome [8, 10, 20–23]: Kopfschmerzen, Schwindel, Orthostasereaktionen, persistierende Rhinitis, Sicca-Symptomatik, verminderter Appetit, Schwitzen, intermittierende (sub-) febrile Körpertemperatur, Durchfall, Haarausfall, Konzentrations- und Gedächtnisstörungen, Palpitationen und Herzrasen, depressive Verstimmung, Hautausschläge. Mit möglichen weiteren noch nicht beschriebenen Symptomen ist zu rechnen.

## 7. Pathomechanismen – was ist bekannt

Die konkrete Pathogenese des Long COVID-Syndroms ist derzeit noch nicht geklärt, sie ist jedenfalls multifaktoriell und wohl auch nicht bei allen Personen ident. In Erwägung gezogene Auslöser sind langdauernde Gewebeschäden, eine Persistenz von Viren oder zumindest Virusbestandteilen sowie eine chronische (Hyper‑/Auto‑) Inflammation.

*Wichtig erscheint hier zumindest eine* **Abgrenzung** *zu:*

1. Symptomen bedingt durch eine persistierende (chronische) Entzündung
2. Folgen eines konkreten Organschadens (z. B. akute Lungen- oder Nierenschädigung)
3. unspezifischen Folgen der Hospitalisation und sozialen Isolation (von ernährungsbedingter Anämie bis hin zum Muskelabbau).

Auch bedürfen alle in Folge geschilderten Symptome unabhängig von einer durchgemachten COVID‑19 einer Klärung, wie in Kap. 10 beschrieben.

Sind eindeutige Erklärungen für residuale Symptome wie Fatigue nicht zu finden, dann können Veränderungen des Stoffwechsels, des Hormonhaushalts, gegen den eigenen Körper gerichtete Entzündungsbotenstoffe und Veränderungen der Hirnfunktion eventuell einen Teil des Leidens erklären.

Eine verminderte **Aktivität der Stresshormonachse** [24] könnte eine gewisse Erschöpfung erklären, denn niedrige Stresshormon-Level können einerseits dazu führen, dass Entzündungsreaktionen nicht gebremst werden und andererseits niedrigen Blutdruck und Kreislaufbeschwerden verursachen.

Auch **Entzündungsmediatoren** spielen vermutlich eine Rolle. So waren im Blut von Menschen, die nach einer Virusinfektion eine chronische Fatigue entwickeln, in der Akutphase Interleukin‑6 und -10 stärker erhöht [25]. Im Körper könnte also noch immer eine Entzündung schwelen.

Gegenwärtig wird unter anderem auch die Hypothese formuliert, pro-inflammatorische Zytokine (Interferon Gamma, Interleukin 7) könnten in der post-infektiösen Phase die Blut-Hirn-Schranke passieren und **autonome Dysfunktionen** verursachen, die sich in einer Dysregulation des Schlaf‑/Wachrhythmus, kognitiver Dysfunktion sowie Müdigkeit und Antriebslosigkeit manifestieren können [26].

Eine weitere Hypothese lautet, dass ein Post-COVID-Syndrom mit einer chronischen subklinischen systemischen Entzündung (Inflammation) einhergehen könnte, wie dies im Alterungsprozess (Aging) beobachtbar ist. Dieses **„Inflammaging“** hätte das Potenzial, bestehende Komorbiditäten zu verschlechtern und altersabhängige Probleme deutlich zu verstärken [27].

Die als **„Zytokinsturm“** bezeichnete schwere, systemische Inflammation ist in allen Altersgruppen beobachtbar, vielfach wurde bei Kindern eine schwere Multisystem-Inflammation mit Ähnlichkeiten zum Kawasaki-Syndrom beschrieben. Hält eine Entzündungsreaktion jedoch über lange Zeit an, so wird angenommen, dass dies zu zellulärer Seneszenz mit Hemmung der Zellproliferation und Resistenz gegenüber Apoptose führt [27].

### 7.1. Chronische Entzündung

Analog zu Autoimmunerkrankungen wird bei Personen mit längeren Beschwerden nach COVID‑19 eine Dominanz des weiblichen Geschlechtes beobachtet und eine T‑ und B‑Zell-Dysregulation (‑dysfunktion) in der Pathophysiologie von Long COVID angenommen [27]. Diskutiert wird, dass das SARS-CoV‑2 in Antigen-präsentierenden Zellen eine „bystander-Aktivierung“ von T‑Zellen gegen Autoantigene auslösen könnte [28]. Mögliche alternative oder weitere Ursache für eine Hyperinflammation könnten Veränderungen der Mikrobiota des Gastrointestinaltraktes [29] bzw. eine Dysbiose [30, 31] sein.

Neben einer T‑Zell-vermittelten Autoimmunität finden sich auch Beobachtungen von anti-Phospholipid-Autoantikörper [32] ebenso wie Auto-Antikörper gegen Zellkerne, Neutrophile, Interferone oder Citrullinpeptide. Derzeit wissen wir nur, dass solche Autoantikörper in der Pathogenese unterschiedlicher Autoimmunerkrankungen (SLE, rheumatoide Arthritis, Sjögren-Syndrom, usw.) eine Rolle spielen [33, 34]. Bei schwerer Erkrankung findet sich regelmäßig eine Lymphopenie [35]. Für einen solchen T‑ bzw. B‑Zellmangel wurde auch ein Zusammenhang mit einem persistierenden Virusshedding nachgewiesen [36, 37]. Untersuchungen zeigen eine im Median gut 50 Tage persistierende Lymphopenie. Erhöhte CRP- bzw. D‑Dimer-Werte finden sich solchen Studien zufolge bei 7,3 %, bzw. 9,5 % der „Genesenen“ bzw. nach Abklingen der Akutphase [38, 39]. Allerdings liegen auch Untersuchungen vor, die keine typischen bzw. verlässlichen Veränderungen von Laborparametern nachweisen [40].

Viele Intensivpatienten verzeichnen bereits bekannte, als **„Post Intensive Care Syndrome“ (PICS)** bezeichnete, Beschwerden. PICS manifestiert sich dabei durch physische, psychische und kognitive Einschränkungen, die sich in einem relevanten Ausmaß nicht vollständig zurückbilden [41].

### 7.2. Persistenz von Viren bzw. Virusbestandteilen

Eine Viruspersistenz für mehrere Monate war nachweisbar [42–44]. Dies trifft offensichtlich besonders auf Personen mit Immundefekten zu [45]. Andere Untersuchungen belegen ein Virusshedding im Respirationstrakt oder Gastrointestinaltrakt für bis zu vier Monaten, wobei Betroffene eine gewisse Immunaktivierung erfahren, aber nicht zwangsläufig unter Symptomen leiden müssen [46, 47].

### 7.3. Spezielle Aspekte

#### 7.3.1. Pathophysiologie der COVID-19 Riechstörung

Da die olfaktorischen Rezeptorneurone selbst kaum ACE-Rezeptoren besitzen, scheint die COVID‑19 bedingte Riechstörung durch eine virusvermittelte Schädigung der Stützzellen der Riechschleimhaut zu entstehen, wodurch die oft nur kurzfristige Störung zu erklären wäre [48]. Eine schwerwiegendere Infektion könnte aber auch die bestehenden Riechnervenzellen irreversibel schädigen. Dadurch braucht die Regeneration des Riechvermögens, die von den Basalzellen der Riechschleimhaut ausgeht, zumindest mehrere Monate. Auch eine Schädigung von Riechzentren im Gehirn (z. B. Bulbus olfactorius) sind denkbar, jedoch als weniger wahrscheinlich anzunehmen [49].

- Die COVID‑19 bedingte Riechstörung entsteht durch eine Schädigung der Stützzellen der Riechschleimhaut.
- Bei Schädigung der Riechnervenzellen kommt es zu einer langfristigen, evtl. dauerhaften Störung.
- Eine zentrale Schädigung (z. B. Bulbus olfactorius) gilt als weniger wahrscheinlich.

## 8. Organsysteme – Übersicht: Leitsymptome und Krankheitsbilder

### 8.1. Pneumologie/Infektiologie

#### 8.1.1. Pneumologische Leitsymptome im Zusammenhang mit Long COVID

##### Dyspnoe (s. a. Abschn. 10.4./12.4.1.)

- Dyspnoe im Rahmen von Long COVID äußert sich vor allem als **Kurzatmigkeit bei Belastung**, und findet sich häufiger nach schwerem Verlauf (nach 3 Monaten noch in ca. 40 %) [50], aber auch nach nicht-hospitalisiertem Verlauf (in ca. 10 %) [10]. Eine milde Dyspnoe über einige Wochen nach der Akuterkrankung wird häufig berichtet. Wenn diese aber nach der Infektion akut neu aufgetreten, zunehmend, oder mehr als nur milde ist, wenn sie den Alltag einschränkt, oder mit weiteren Symptomen einhergeht, erfolgt die differenzialdiagnostische Abklärung

##### Husten (s. a. Abschn. 10.5.)

- Husten nach akuter Erkrankung findet sich häufig, z. B. noch in 17 % nach 3 Monaten [50]. Bei persistierendem Husten ist leitliniengemäß eine Abgrenzung zu nicht pneumologischen Hustenursachen zu empfehlen, bzw. die weiterführende Diagnostik wie bei jedem anderen Husten.

##### Fieber

- Rezidiverende Infektionen: Sekundäre bakterielle, virale oder fungale Infektionen nach COVID v. a. nach SARS-CoV2 assoziierter Lungeninfektion
- Das Ausmaß einer persistierenden Immunsuppression und einer dadurch bedingten erhöhten Infektionsanfälligkeit (wie bei Masern) ist für COVID‑19 noch nicht ausreichend untersucht

##### Thorakale Schmerzen (s. a. Abschn. 10.6.)

- Thorakale Beschwerden treten häufig bei Patient:innen noch Wochen nach akuter Infektion auf. Die Ätiologie ist unklar, möglicherweise Folge der suszipierten autonomen Dysfunktion und Muskelschwäche im Rahmen von Long COVID bzw. des Post-COVID‑19 Syndroms.
  Beispielsweise gibt es bei physiotherapeutischen Untersuchungen Hinweise für eine Einschränkung der Zwerchfellmobilität sowie Hinweise auf eine Muskelschwäche der Atemmuskulatur [51].

#### 8.1.2. Krankheitsbilder mit möglicher Assoziation zu Long COVID

##### Residuale Pneumonie

Im Verlauf bis zu 100 Tage nach COVID‑19 Beginn bessern sich bei 2/3 der Patient:innen mit Viruspneumonie die CT-Auffälligkeiten deutlich und es zeigen sich nur geringe Residuen (Milchglas und Retikulationen) [50]. Eine fehlende Besserung bzw. Zeichen einer akuten oder rezidivierenden Infektion bedürfen einer spezifischen Abklärung. Auch longitudinale Daten einer chinesischen Kohorte bis 6 Monate nach COVID‑19 zeigen in 2/3 der Patient:innen eine deutliche Besserung (38 % komplette Resolution, geringe Residuen in 27 %), aber in 35 % Fibrose-ähnliche Veränderungen, vor allem nach ARDS, längerem Krankenhausaufenthalt und höherem Alter [52]. In der bisher einzigen publizierten 12-Montasstudie einer weiteren chinesischen Kohorte verbleiben nach 12 Monaten in 24 % radiologische Veränderungen, vor allem bei jenen mit ausgeprägten Veränderungen während der Hospitalisation [53].

##### Pulmonalembolie

Trotz hoher Embolierate bei kritischem Verlauf auf der Intensivstation zeigen Nachsorge-Studien klinisch eine geringe Inzidenz für Pulmonalembolien [1, 50]. Allerdings wurden das Vorliegen von Embolien oder Mikroembolien hierbei nicht systematisch untersucht. Die Frequenz, klinische Bedeutung und therapeutische Konsequenz von möglicherweise noch bestehenden Mikroembolien (bzw. „Microvascular Injury“) ist noch nicht geklärt [54].

- Bei akuter Dyspnoe mit D‑Dimer Erhöhung oder anhaltender Dyspnoe mit Belastungsdesaturation oder Zeichen einer pulmonalen Hypertonie oder nur geringen strukturellen Veränderungen (unverhältnismäßig zur Dyspnoe) ist ein Angio-CT indiziert.
- Ein regelmäßiges Screening nach Mikroembolien ist in der Routine nicht empfohlen.

##### Lungenfibrosen

Ob und wie oft es zu einer progressiven Fibrosierung der Lunge kommt ist bis dato unklar [55]. Bei Befunden, die für einen progressiven interstitiellen Prozess sprechen, sollte eine weitere Abklärung mittels Bronchoskopie mit BAL und Biopsie folgen – entsprechend den Empfehlungen zur Diagnostik von interstitiellen Lungenerkrankungen.

##### Atemmuskelschwäche

Die muskuloskelettale Beteiligung bei Long COVID hat einen Gewichtsverlust und somit einen Muskelverlust zur Folge. Damit verbunden kann eine Atemmuskelschwäche als Grundlage der Dyspnoe vorliegen (PI_max_ < 80 mbar bei Männern, < 70 mbar bei Frauen).

##### Schlafassoziierte Störungen

Einschlafstörungen sollten v. a. bei Fatigue abgefragt werden, da eine Schlafhygiene die Fatigue verbessern kann. Bei Durchschlafstörungen kann ein Schlafscreening oder eine Polysomnographie erfolgen, um diese zuzuordnen [56].

#### 8.1.3. Methoden der pneumologischen Abklärung

- In Ruhe (Spirometrie, Bodyplethysmographie, Diffusionskapazität, Blutgasanalyse, maximale inspiratorische Atemmuskelkraftmessung (MIP oder PI_max_)) und
- unter Belastung (z. B. 1 Minute-Sit to Stand Test, 6‑Minuten Gehtest, Spiro‑/Ergometrie),

unter Berücksichtigung möglicher Vorerkrankungen sollte bei

- pathologischer Lungenfunktion (FVC, TLC) oder
- pathologischem Blutgasbefund (SpO2 in Ruhe oder Belastung) oder
- einer verminderten CO-Diffusionskapazität (DLCO) eine Bildgebung mittels HRCT durchgeführt werden. Bisherige Studien zeigen, dass eine eingeschränkte Diffusionskapazität (DLCO) in der COVID‑19 Nachsorge von hospitalisierten Patienten in ca. 25 % diagnostiziert wird [1, 50].

Weiterführende Bildgebung: Das häufigste bildgebende Korrelat im HRCT nach einer Viruspneumonie sind Milchglastrübungen und Konsolidierungen, gefolgt von linearen Verdichtungen, sowie in Einzelfällen Traktionsbronchiektasen und lokalisierte fibrotische Zeichen [1, 50, 57].

### 8.2. Kardiologie

#### 8.2.1. Allgemeines

Eine kardiale Beteiligung bei Long COVID ist nicht selten. In einer Studie an 201 Personen mittleren Alters, die COVID‑19 durchgemacht hatten (meist ohne Hospitalisierung) und persistierende Symptome aufwiesen, wurde etwa 4,5 Monate nach der Erkrankung eine Multi-Organ MR Untersuchung durchgeführt. Bei 26 % der Patienten zeigten sich (meist milde) myokardiale Veränderungen: Myokarditis in 19 %, systolische Dysfunktion in 9 % [58].

Besonders bei kardialen Vorerkrankungen sind Verschlechterungen nicht selten. Die Folgen dieser akuten kardialen Manifestationen können auch bei Long COVID eine Rolle spielen. Eine Aufzählung dieser Akutereignisse findet sich weiter unten [59].

Des Weiteren wurde gezeigt, dass kardiovaskuläre Komplikationen innerhalb der ersten 6 Monate nach einer COVID‑19 deutlich erhöht sind. Dabei scheint die Inzidenz dieser direkt mit dem Schweregrad der vorangegangenen Erkrankung assoziiert zu sein. Patient:innen, die während ihrer akuten Erkrankung hospitalisiert waren, haben ein doppelt so hohes Risiko im weiteren Verlauf auch eine kardiale Komplikation zu entwickeln wie nicht Hospitalisierte. Hierbei ist insbesondere an venöse Thrombosen, ischämische Schlaganfälle, Myokardinfarkte, Lungenembolien und auch das Auftreten einer Herzinsuffizienz zu denken (siehe dazu AWMF S1-Leitlinie – Post-COVID/Long-COVID [60]).

#### 8.2.2. Kardiologische Leitsymptome im Zusammenhang mit COVID-19

##### Dyspnoe und eingeschränkte Leistungsfähigkeit (s. a. Abschn. 10.2., 10.4./12.2., 12.4.1.)

In einer Long COVID-Population wurde Dyspnoe von 43,4 % aller Patienten angegeben. Die Dyspnoe ist sehr unspezifisch, jedoch eines der häufigsten Symptome in der Kardiologie. Wenn eine Herzinsuffizienz zugrunde liegt, wird anhand der NYHA-Klassifizierung in das NYHA-Stadium I–IV eingeteilt [12].

##### Thorakale Schmerzen (s. a. Abschn. 10.6.)

Ca. 21,7 % der Patienten nach COVID‑19 präsentieren sich mit thorakaler Schmerzsymptomatik [59].

##### Palpitationen

Die Häufigkeit des Auftretens von Palpitationen bei Long COVID wurde bis dato noch nicht beschrieben. Zur Abklärung dieser Beschwerden empfiehlt sich wie sonst auch die Durchführung eines 12 Kanal-EKG, eines Holter-EKG sowie eine Ergometrie.

##### Kreislauflabilität (s. a. Abschn. 10.8., 10.9./12.2., 12.4.5.)

Typische klinische Erscheinungsformen des Postural Tachykardia Syndroms (POTS) [61]:

- Orthostaseintoleranz
- Tachykardie bei Orthostase
- Palpitationen
- Schwindelgefühl („dizziness“)
- Sehstörungen
- Präsynkopen
- Belastungsintoleranz

Dieses wird häufig durch eine Virusinfektion ausgelöst und passt gut in den Long COVID-Formenkreis. Einen guten Hinweis gibt die Blutdruckmessung im Stehen (auch bei Selbstmessungen!) und der Schellong-Test. Die Kipptischuntersuchung sichert die Diagnose, ist aber nur an wenigen Abteilungen durchführbar und daher besonderen Fällen vorbehalten.

Zur weiteren Differenzialdiagnostik s. Abschn. 10.9.

#### 8.2.3. Weitere kardiale Krankheitsbilder im Zusammenhang mit COVID-19

Mögliche kardiale Begleiterscheinungen der akuten COVID‑19, deren Auswirkungen auch bei Long COVID eine Rolle spielen können, sind umfangreich und umfassen u. a. [59]:

- Akute Perikarditis
- Beschwerden ohne spezifische Ätiologie wie Palpitationen, Kreislauflabilität (s. a. Abschn. 10.8., 10.9.)
- Akute Herzinsuffizienz bis zum Lungenödem
- Akutes Koronarsyndrom (NSTEMI, STEMI)
- Akute Stresskardiomyopathie
- Akute Myokarditis
- Supraventrikuläre und ventrikuläre Arrhythmien (am häufigsten Vorhofflimmern)
- Akute rechtsventrikuläre Dysfunktion (nicht nur bei Lungenembolie)

#### 8.2.4. Methoden der kardiologischen Abklärung

- Die physikalische Untersuchung dient der Erkennung von Zeichen einer hydropischen Dekompensation und umfasst auch die Blutdruckmessung.
- Mittels 12 Kanal-EKG werden Frequenz und Rhythmus sowie allfällige Rhythmusstörungen erfasst. Unspezifische Veränderungen können bereits auf eine Herzinsuffizienz oder eine KHK hinweisen.
- Eine Laboruntersuchung zum Ausschluss anderer internistischer Ursachen für Dyspnoe soll bei (klinischen oder anamnestischen) Hinweisen auf Herzinsuffizienz bereits die Bestimmung eines NTproBNP inkludieren. Ein NTproBNP Wert < 125 pg/ml schließt das Vorhandensein einer symptomatischen Herzinsuffizienz weitgehend aus [62].
- Die Echokardiographie ist beweisend für die Diagnostik von verschiedenen Formen der Herzinsuffizienz (HFpEF bis HFrEF), wegweisend für die Erfassung einer pulmonal-arteriellen Hypertension und liefert Hinweise auf eine KHK (z. B. Narben nach abgelaufenem Herzinfarkt).
- Belastungsergometrie – diese hat aufgrund der geringen Sensitivität im diagnostischen Algorithmus zur Abklärung einer koronaren Herzkrankheit mittlerweile einen geringeren Stellenwert. Sie wird aufgrund der guten Verfügbarkeit als Vorfelddiagnostik aber immer noch häufig eingesetzt. Sollte sich der Verdacht auf das Vorliegen einer koronaren Herzerkrankung erhärten, kommen je nach Höhe der Vortestwahrscheinlichkeit weitere nicht invasive Untersuchungsmethoden (Myokardszintigraphie, Stressechokardiographie, Koronar-CT) oder die Koronarangiographie zur Anwendung. Im Rahmen von Long COVID ist die Objektivierung einer Leistungseinschränkung ein Vorteil der Ergometrie.
- Der nicht-invasive Gold-Standard für die Diagnose einer Myokarditis ist die Kernspintomographie, die in kleinen Fallserien nicht selten Myokarditis-typische Veränderungen nach COVID‑19 zeigte [59].
- Kipptischuntersuchung bei orthostatischen Beschwerden

### 8.3. Neurologie

#### 8.3.1. Allgemeines

SARS-CoV2 konnte in unterschiedlichen Strukturen des Gehirnes nachgewiesen werden [63]. Die Viren verursachen nur sehr selten eine Enzephalitis [64]. Die Bedeutung dieser Befunde – insbesondere für Langzeitfolgen – ist aktuell sowohl für die Struktur als auch die Funktion des Gehirns unklar. Hier bedarf es weiterer grundlagenwissenschaftlicher und klinischer Studien.

Bei einer prospektiven Dokumentation von spitalspflichtigen COVID‑19 Patient:innen aus New York wird berichtet, dass **nur 13** **% eine neue neurologische Erkrankung** zeigten, die auch vom Facharzt für Neurologie bestätigt wurde. Am häufigsten war hier die Enzephalopathie als Folge der systemischen Entzündungsreaktion bei der SARS-Cov2-Infektion, die dem bekannten Krankheitsbild septischer Enzephalopathien entspricht [65].

Über Folgen im Bereich von Neurologie und Psychiatrie berichtete **fast jeder zweite Patient** Müdigkeit, Muskelschmerzen, Biorhythmusstörungen, Angst oder Depression. Es zeigte sich in einigen Studien eine positive Korrelation von Schweregrad der COVID-Erkrankung zu den Folgezuständen [1].

#### 8.3.2. Neurologische Leitsymptome von Long COVID

- Postinfektiöse Müdigkeit
- Hirnleistungsstörungen („brain fog“)
- Konzentrationsstörung
- Gedächtnisstörung
- Schlafstörungen
- Extremitätenschmerz (myalgisch, neuropathisch)
- Sensibilitätsstörungen (u. a. Missempfindungen, Taubheit)

Eine Vielzahl anderer (u. a. Hyp- und Anosmie, Schwindel/Benommenheit, Depression, Angst), seltener (u. a. Kopfschmerzen, autonome Dysfunktionen) und teilweise schlecht definierter (u. a. Fatigue) Symtome wurden berichtet. Ob die häufig und konsistent berichtete Fatigue mit dem schlecht definierten und wissenschaftlich umstrittenen „Myalgic encephalomyelitis Syndrom“ (ME/CFS) ätiologisch verglichen werden kann ist fraglich. Die DGN hat mit Jänner 2021 eine lebende S1-Leitlinie zur Beschreibung von neurologischen Komplikationen para- und postinfektiös von COVID‑19 veröffentlicht [66]. Ob es postinfektiösen Morbus Parkinson – in Analogie zur Encephalitis lethargica gibt, ist nicht belegt. Parainfektiöse immunologische Erkrankungen des Nervensystems wie ADEM (Akute demyelinisierende Enzephalomyelitis) und Guillain Barré Syndrom wurden beschrieben [67, 68].

#### 8.3.3. Neurologische Krankheitsbilder im Rahmen von Long COVID

##### Postinfektiöse Müdigkeit (s. a. Abschn. 10.1., Kap. 12)

Die meist als „Fatigue“ bezeichnete postinfektiöse Müdigkeit ist eines der Schlüsselsymptome von COVID‑19 und tritt in der Akutphase bei bis zu 95 % der Erkrankten auf. Auch drei Monate nach der Erkrankung klagten in einer Studie mehr als 80 % der Betroffenen über eine chronische Erschöpfung. Pathophysiologisch ist dieses Bild nicht gut verstanden. Fatigue wurde auch nach leichten Krankheitsverläufen berichtet. Eine postinfektiöse Müdigkeit bildet sich dennoch meist spontan zurück. Eine kausale Therapie steht nicht zur Verfügung. Mehr dazu s. Abschn. 9.1.

##### Störungen der Hirnleistung („brain fog“) (s. a. Abschn. 10.7., Kap. 12)

Fieber und Allgemeinerkrankungen können zu einer beeinträchtigen Hirnfunktion in individuell unterschiedlichem Ausmaß führen. Konzentrationsschwäche, Antriebsminderung, reduzierte Merkfähigkeit und Kopfschmerzen bis hin zum Delir sind typische Manifestationen der akuten Phase. Wenn solche Beschwerden nach der Genesung persistieren und die Grunderkrankung COVID‑19 war, wird heute oft in der Literatur von „brain fog“ berichtet. Die Bewertung der wissenschaftlichen Evidenz ist in diesem Bereich besonders schwierig, weil die Hirnfunktion komplex ist und zahlreichen Einflussfaktoren unterliegt.

Pathophysiologisch hat sich eine Gruppe aus Freiburg mittels FDG-PET-Untersuchung des Gehirns mit dieser Frage auseinandergesetzt. Mit der [18F]Fluordesoxyglucose-Positronenemmissions-Tomografie wurde in einem kleinen Patientenkollektiv eine Verminderung des Glukosestoffwechsels im Gehirn nachgewiesen, die mit solchen kognitiven Defiziten assoziiert ist [69].

Die Arbeitsgruppe publizierte bereits Ergebnisse eines Follow-up von acht Patienten der Originalstudie. Im Verlauf kam es zu einer signifikanten Besserung der kognitiven Defizite, wenngleich einige Betroffene auch sechs Monate nach der Akuterkrankung noch kein Normalniveau erreicht hatten.

Die Symptomverbesserung ging mit einer weitgehenden Normalisierung des Hirnstoffwechsels einher. Die kognitiven Beeinträchtigungen korrelierten also mit dem Grad der Verminderung des Glukosemetabolismus, so dass dieser im Einzelfall als Biomarker für kognitive Post-COVID-Symptome herangezogen werden könnte.

Gesicherte Therapien für Hirnleistungsstörungen bei Long COVID existieren nicht. Von einer spontanen Besserung ist auszugehen. Ob sich z. B. eine Alzheimerdemenz oder andere neurodegenerativen Erkrankung als Folge einer COVID‑19 manifestieren können, wird diskutiert und bedarf weiterer klinischer Studien. Bei Persistenz länger als 3 Monate ist eine fachärztliche Untersuchung durch Neurologen zu empfehlen.

##### Schmerzen Myalgien (s. a. Abschn. 10.11./11.4.6.)

Muskelschmerzen treten bei SARS-CoV2-Infektion oft im Akutstadium auf, können aber auch nachher über Monate persistieren. Die Pathogenese ist nicht geklärt, die Differentialdiagnose ist umfangreich und wesentlich. Zur Klärung tragen vor allem die Anamnese und der klinische Status bei, apparative Zusatzuntersuchungen sind im Einzelfall sinnvoll. In einer spanischen Case-Control Studie von hospitalisierten COVID‑19 Patienten zeigte sich sieben Monate nach Krankheitsbeginn, dass das Auftreten von Myalgie bei Hospitalisierung mit präexistenten muskuloskelettalen Beschwerden korrelierte. Weiters war Myalgie bei Hospitalisierung ein Prädiktor für die längerfristige Persistenz von Muskelschmerzen.

##### Neuropathische Schmerzen (s. a. Abschn. 10.11./11.4.6.)

Neuropathische Schmerzen werden nur vereinzelt berichtet und sind insbesondere vom Muskelschmerz abzugrenzen.

#### 8.3.4. Abgrenzung anderer Beschwerdebilder gegenüber Long COVID

- **Critical illness Neuromyopathie** – prolongierte Intensivaufenthalte mit Multi-Organ-Versagen führen zu einer nutritiv-toxisch bedingten Involution von Skelettmuskulatur und peripheren Nerven. Dieses Zustandsbild ist seit Jahrzehnten bekannt und wird anamnestisch, klinisch und elektrophysiologisch diagnostiziert.
- Persistenz einer **septisch-toxisch-metabolischen Enzephalopathie** nach ICU – Vor allem bei subklinischen zerebralen Vorschäden (z. B. Altersveränderungen des Gehirns) kann eine schwere Infektion mit ICU-Behandlungsbedarf durch Ausschüttung von Entzündungsmediatoren, Toxinen und Neurotransmitter-Imbalance zu einer prolongierten Aufwachphase mit Delir und persistierenden kognitiven Einbußen führen. Dieses Zustandsbild ist seit Jahrzehnten bekannt und wird anamnestisch, klinisch und mithilfe anderer Zusatzuntersuchungen diagnostiziert.
- **Verschlechterung vorbestehender neurologischer Erkrankungen** – Alle Erkrankungen des zentralen oder peripheren Nervensystems sowie der Skelettmuskulatur können sich durch eine schwere Allgemeinerkrankung passager oder auch dauerhaft verschlechtern. Patient:innen erreichen nach der Genesung von der Allgemeinerkrankung nicht mehr den vorherigen funktionellen Status. Dieses Zustandsbild ist seit Jahrzehnten bekannt und wird anamnestisch und klinisch diagnostiziert.
- Klinische **Manifestation subklinischer Gehirnerkrankungen** durch COVID‑19 (z. B. Mild Cognitive Impairment) – Chronische und bis dato unerkannte und subklinische Vorschädigungen des Gehirns können durch eine akute Infektion funktionell dekompensieren und nach Ausheilung des Infektes sich klinisch „erstmanifestieren“. Dieses Zustandsbild bedarf einer fachärztlichen Abklärung nach state of the art.

#### 8.3.5. Methoden der neurologischen Abklärung

- Fokussierter neurologischer Status (Motorik, Sensibilität, kognitive Funktion)
- Labor: zur gezielten (!) Differenzialdiagnostik entspr. Anamnese und Klinik, z. B. zur Identifikation entzündlicher Erkrankungen oder ursächlicher Stoffwechselstörungen: CK, Differenzialblutbild + Gerinnung, Blutsenkung (BSG) und CRP als Hinweise auf Infekte sowie eine autoimmune Genese, ggf. auch Myoglobin, Leberenzyme, Elektrolyten (Na, K, Ca).
- Zur Beurteilung der kognitiven Leistungsfähigkeit kann bereits in der Hausarztpraxis ein MMSE (Minimental State Examination) oder MoCA (Montreal Cognitive Assessment) orientierend durchgeführt werden, ist aber für enzephalopatische Störungen nicht validiert.
- Weiterführende Untersuchungen z. B. zB. MRT, EMG/ENG, autonome Testbatterie/Kipptisch, Geruchstests, Neuropsychologische Untersuchung, Schlaflabor, Neuropsychosomatik.

Weitere Quellen: [1, 3, 70–77].

### 8.4. Hals-Nasen-Ohrenheilkunde

#### 8.4.1. Allgemeines

Nach COVID‑19 zeigen Patient:innen signifikante Beeinträchtigungen von Geruchssinn, Atmung, Stimme und Schlucken, die in einer individualisierten Rehabilitation nach COVID‑19 Berücksichtigung finden müssen und einer HNO-ärztlich/phoniatrischen Diagnostik und logopädischen Therapie bedürfen.

#### 8.4.2. Leitsymptome und Krankheitsbilder im HNO-Bereich mit Assoziation zu COVID-19

##### Riech- und Schmeckstörungen (s. a. Abschn. 10.3./12.4.4.)

Riechstörungen stellen ein häufiges Symptom der Infektion mit SARS-Co-V2 dar [78]. 60–80 % der Betroffenen klagen über einen Verlust des Riech- und Schmeckvermögens, oft nur vorübergehend für wenige Tage bis Wochen, eine Persistenz ist jedoch auch über mehrere Monate möglich [79]. Die Riechstörung wird aufgrund des plötzlichen Auftretens meist von den Patient:innen deutlich wahrgenommen. Die direkte Assoziation zu COVID‑19 im Unterschied zu vorbestehenden Einschränkungen sollte gesichert sein. Untersuchungen des Langzeitverlaufs COVID‑19 bedingter Riechstörungen zeigen, dass auch ein Riechverlust bis zu einem Jahr und darüber hinaus, vorkommen. Folglich kommt es bei den Betroffenen zu einer deutlichen Einschränkung der Lebensqualität und dem Wunsch nach Therapie der Beschwerden [80].

Besonders beeinträchtigend ist die **Parosmie** (Fehlriechen, die veränderte Wahrnehmung von Gerüchen, die meist als unangenehm wahrgenommen werden). Diese Form der Riechstörung tritt bei vielen Betroffenen mehrere Wochen bis Monate nach initialem Verlust des Riechvermögens auf, nachdem bereits ein Teil des Riechvermögens zurückgekehrt ist. Studien weisen darauf hin, dass dies als Zeichen der Regeneration des Geruchssinns aufgefasst werden kann [81].

##### Andere Ursachen von Riechstörungen

Ein vermindertes Riechvermögen kann prinzipiell auf zwei pathophysiologische Mechanismen zurückgeführt werden. Zum einen kommt es bei konduktiven Riechstörungen (z. B. bei Nasenpolypen) zu einer verminderten Zuleitung der Duftstoffe zur Riechschleimhaut im oberen Bereich der Nase.

Bei den sensori-neuralen Riechstörungen liegt die Ursache entweder in einer Funktionsstörung der Riechsinneszellen oder in übergeordneten zentralnervösen Strukturen entlang der Riechbahn.

Die COVID‑19 bedingte Riechstörung fällt in die zweite Gruppe, in die auch vor Beginn der COVID‑19-Pandemie andere Viren (z. B. Influenza‑, Parainfluenza‑, Rhinoviren) unter dem Begriff der post-infektiösen bzw. post-viralen Riechstörung zusammengefasst wurden [82].

Ebenfalls in die Gruppe der sensori-neuralen Riechstörungen fallen die post-traumatischen Riechstörungen, der Riechverlust bei neurologischen oder neurodegenerativen Erkrankungen (z. B. M. Parkinson, M. Alzheimer, Insult), bei medikamentös-toxischen Einflüssen, bei Tumoren der vorderen Schädelbasis, nach Chemo- oder Strahlentherapie, oder bei internistischen Erkrankungen (z. B. Leber‑, Nierenerkrankungen). Selten besteht eine kongenitale Anosmie (isoliert oder im Rahmen des Kallmann-Syndroms).

Sollte keine Ursache im Rahmen der HNO-Abklärung, neurologischen, internistischen und allgemeinmedizinischen Abklärung gefunden werden, liegt eine idiopathische Riechstörung vor (bis zu 15–20 % der Patienten von Spezialambulanzen). Hier wird die Durchführung einer MRT-Untersuchung des Schädels empfohlen [82].

Als wichtige Differentialdiagnose jeder Riechstörung ist die Mischform einer sensori-neuralen und konduktiven Riechstörung in Form der chronischen Rhinosinusitis mit und ohne Nasenpolypen in Betracht zu ziehen. Sollten anamnestisch Hinweise auf diese Erkrankung vorliegen (z. B. Nasenatmungsbehinderung, Druckgefühl im Gesicht, nasale Sekretion), wird eine HNO-ärztliche Abklärung empfohlen.

##### Stimm- und Schluckprobleme

**Oropharyngeale Dysphagien** können u. a. nach Langzeitintubation, -beatmung, Tracheostomie sowie Intensivpflege sowohl bei NON-COVID‑19- als auch COVID‑19-Patient:innen auftreten. Pathophysiologische liegen möglicherweise eine Koordinationsstörung zwischen Atmung und Schlucken, Pharynxschwäche, ein inkompletter laryngealer Verschluss oder eine Critical Illness Polyneuropathie zugrunde. Ob eine COVID‑19 spezifische, neurogene Dysphagie-Ätiologie vorliegt, ist dzt. nicht zu differenzieren und Gegenstand von Untersuchungen. Bei COVID‑19 Patient:innen zusätzlich beeinträchtigend ist potenziell die Bauchlagerung mit verminderter Zugangsmöglichkeit im Rahmen der oralen Hygiene und potenziell vermehrter bakterieller Kolonisation der Mundhöhle mit denkbar erhöhtem Aspirationsrisiko.

Die Früherkennung einer Dysphagie ist für ein adäquates Patient:innen-Management wesentlich.

Bei 27 % der Patient:innen mit milder bis moderater COVID‑19 wurde eine **Dysphonie** beobachtet. Sie kann als Initialsymptom (19 %), nach Erkrankung – selbst bei ursprünglich nicht hospitalisierten Patient:innen – oder im Rahmen von Long COVID auftreten und zu verbalen Kommunikationsproblemen führen. Dysphonie nach COVID‑19 kann einerseits den obengenannten unspezifischen und den fraglich COVID‑19 spezifischen pathophysiologischen Mechanismen geschuldet sein (Intubationsschäden am Kehlkopf, Folgen der Langzeitintubation oder Störung der neurogenen Koordination). Überdies bestärkt eine fraglich höhere Expression von ACE 2 bei COVID‑19 die Hypothese von gesteigerten entzündlichen Prozessen der Stimmlippen („Corditis-Ätiologie“), wobei geschlechtsspezifische Unterschiede diskutiert werden. Darüber hinaus wurden auch paradoxe Stimmlippenbewegungen beobachtet. Außerdem kann eine Atemstörung nach COVID‑19 auch laryngeal bedingt sein.

Weitere Quellen: [83–93].

##### Hörstörungen

Das Auftreten eines COVID‑19 bedingten Hörverlustes wurde in der Literatur anekdotisch berichtet [94]. Ein zeitlicher Zusammenhang sollte gegeben sein, der entweder am Höhepunkt der Erkrankung oder auch wenige Wochen nach der Infektion zu finden ist. Es kommt entweder zu einer Schädigung des Labyrinths (Hörschnecke und Bogengänge) durch die akute Infektion oder die nachfolgende Immunreaktionen. Neben der Hörstörung ist auf begleitende Symptome einer Labyrinthitis wie Schwindel und Tinnitus sowie Nystagmus zu achten.

Andere Ursachen der Hörstörung sind mittels otoskopischer Untersuchung (Cerumen, Otitis externa, Otitis media) oder durch weitere Untersuchungen auszuschließen (z. B. akustisches Trauma, Schädel-Hirn-Trauma, M. Meniere, Otosklerose, Presbyakusis, medikamentös-toxische Ursachen, oder innere Erkrankungen wie Hypertonie, Diabetes mellitus).

Bei unklarem Befund sollte eine retrocochleäre Ursachen der Hörstörung (z. B. Akustikusneurinom) durch ein MRT des Schädels ausgeschlossen werden.

#### 8.4.3. Methoden der Diagnostik im HNO-Bereich

##### Riechtests

Im Rahmen des Managements der Betroffenen hat sich gezeigt, dass die Durchführung von Riechtests einen positiven Effekt aufweist, da dies den Patient:innen vermittelt, dass die Beschwerden ernst genommen werden. Außerdem hat sich gezeigt, dass die subjektive Selbsteinschätzung des Riechvermögens oft nicht mit objektivierenden Testverfahren übereinstimmt [95].

Es sind verschiedene Screening-Tests zur einfachen und schnellen Testung des Riechvermögens erhältlich und auch für die Selbst-Testung geeignet [96]. Bei Notwendigkeit der ausführlichen Testung (z. B. für gutachterliche Fragestellungen) sollten aber idealerweise Tests mehrerer olfaktorischer Dimensionen (Geruchsschwelle, Diskrimination, Identifikation) durchgeführt werden [97, 98]. Nur so kann die individuelle Diagnose einer Anosmie (Verlust des Riechvermögens), Hyposmie (vermindertes Riechvermögen) oder Normosmie (normales Riechvermögen) gestellt werden.

Besonders bei anamnestischen Unklarheiten, ob eine wirkliche Schmeckstörung (auf süß, sauer, salzig, bitter, umami) oder eine Riechstörung vorliegt, hilft die Durchführung validierter Tests der olfaktorischen und/oder gustatorischen Sensitivität [99]. So klagen die meisten Patient:innen mit Riechstörungen über eine damit einhergehende Störung des Feingeschmacks beim Essen und Trinken (durch das Fehlen der retronasalen Geruchs-Wahrnehmung).

##### Riechtraining

**Figure 2:**
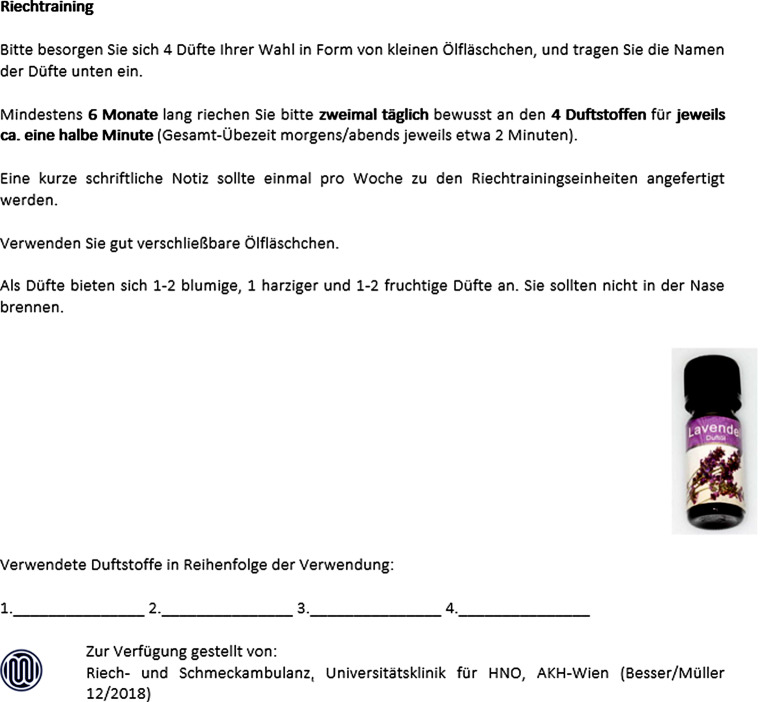

S. dazu Abb. 2 – Patient:inneninformation zur Gestaltung des Riechtrainings.

### 8.5. Dermatologie

#### 8.5.1. Allgemeines

Hautveränderungen können Begleitsymptome von der COVID 19-Infektion sein, sowie bei vielen anderen Virusinfektionen. In der Literatur finden sich derzeit zahlreiche Hypothesen bezüglich der pathophysiologischen Mechanismen. Es gibt jedoch derzeit diesbezüglich keine sichere Evidenz. Weitere Studien sind daher diesbezüglich erforderlich.

Die Inzidenz von Hautmanifestationen wurde auch aufgrund methodischer Mängel der meisten Studien (Selektionsbias) überschätzt.

Es gibt in der Literatur zum Beispiel Berichte von COVID‑19 assoziierten vesikulösen Exanthemen in denen schlussendlich verschiedene Herpesviren mittels PCR nachgewiesen werden konnten.

Der direkte Erregernachweis aus der Haut ist bisher nicht oder nur unzureichend gelungen, insbesondere bei den erwähnten Manifestationen.

COVID-19 assoziierte Hautmanifestationen können von einer sehr polymorphen Natur sein. Derzeit unterscheiden wir nachfolgende klinische Hautbilder, die länger als 4 Wochen anhalten können:

#### 8.5.2. Dermatologische Krankheitsbilder

##### Papulovesikulöses Exanthem

- Häufigkeit: 4–18 % der Hautveränderungen
- Zeitpunkt: Durchschnitt 3 Tage nach Symptombeginn
- Dauer: mediane Dauer 20 Tage, maximale Dauer 70 Tage
- Verteilung:
  1. generalisiertes, polymorphes Muster
  2. lokalisiertes Muster
    Symptomatik: kaum Pruritus
    Schweregrad der COVID‑19-Infektion: moderat

##### Akrale Pernionen, sogenannte COVID-„Toes“

- Häufigkeit: ca. 28 % der Hautmanifestationen
- Zeitpunkt: asymptomatische PatientInnen
- Dauer: mediane Dauer 15 Tage, maximale Dauer länger als 130 Tage, Erkrankungsgruppe: Kinder und junge Erwachsene
- Verteilung: Füße, seltener Hände, Symptomatik: Schmerzen, Brennen, selten Juckreiz
- Schweregrad der COVID 19-Infektion: asymptomatische PatientInnen. In Zusammenhang mit den sogenannten COVID-Zehen wurde nachgewiesen, dass die meisten Patienten negativ im PCR Test sind.

##### Livedo reticularis/racemosa Hautveränderungen

- Häufigkeit: weniger als 4 % der Hautmanifestationen
- Es gibt hierbei zwei Untertypen:
  1. Untertyp: Livedo reticularis Typ-Verteilung:
    Verteilung: symmetrisch, Symptomatik mild
    Schweregrad der COVID 19-Infektion: meist bei mildem Verlauf und nicht assoziiert mit thromboembolischen Ereignissen.
  2. Untertyp: Livedo racemosa Typ-Verteilung:
    Verteilung: größere asymmetrische, anuläre Läsionen
    Schweregrad der COVID 19-Infektion: häufig assoziiert mit schwerer Koagulopathie

##### Purpura/vaskulitische Hautveränderungen

- Häufigkeit: 1–8 % der Hautmanifestationen
- Verteilung: Ausbreitung bis zu nekrotischen, ulzerierenden Läsionen; generalisiert oder lokalisiert im intertriginösen Areal.
- Erkrankungsgruppe: ältere Patient*innen
- Schweregrad der COVID 19-Infektion: schwere COVID‑19-Verläufe
- Die Hautveränderungen sind mit der höchsten Mortalität assoziiert.

##### Immunvermittelte Hauterkrankungen

Durch Infektionen mit SARS-CoV‑2 können generalisierte, immunvermittelte Hauterkrankungen getriggert werden. Folgende dermatologische Erkrankungen im Rahmen von Long COVID sind derzeit in der Literatur beschrieben worden: Psoriasis vulgaris, systemischer Lupus erythematodes, Vaskulitis, Dermatomyositis und chronische rheumatologische Erkrankungen.

##### Para-infektiöse Phänomene

Wie bei jeder Infektionserkrankung können para-infektiöse Phänomene auch bei einer Covid-Infektion auftreten. Ein in der Literatur beschriebenes Beispiel ist Haarausfall.

##### Haarausfall

Auch Haarausfall wird als ein mögliches Long COVID-Symptom in der Literatur beschrieben. Um einen kausalen Zusammenhang oder die Spezifität beurteilen zu können bedarf es noch weiterer Daten. In einer chinesischen Kohorte trat Effluvium in 20 % der Patient*innen Post-COVID auf. Eine spezifische Behandlung ist hier nicht notwendig – eine je nach Haarwuchszyklus (Telogen) zeitverzögerte jedoch vollständige Wiederherstellung sollte auch ohne die Gabe von Haarwuchs-stimulierenden Medikamenten erreicht werden. Eine reversible Ursache (u. a. Schilddrüse, Eisenmangel) sollte ausgeschlossen werden.

##### Seltenes

Hyperästhesien, toxisches Handekzem

#### 8.5.3. Methoden der dermatologischen Abklärung

Nachfolgende Methoden sollen zum Ausschluss von Differentialdiagnosen helfen:

- Hautbiopsie inklusive einer direkten Immunfluoreszenz (nur bei Persistenz und unklarer Diagnose)
- Labors: Blutbild, Nierenfunktionsparameter, Elektrolyte, Leberfunktionsparameter, CRP, Gerinnung, bedarfsweise antinukleäre Antikörper + Subsets, ANCA, C3, C4, cirk. Immunkomplexe und Doppelstrang-DNA.

#### 8.5.4. Differentialdiagnosen

Virusexantheme mit anderen Viren, Arzneimittelexantheme, Vaskulitis anderer Genese.

**Zur Behandlung**: s. Abschn. 12.4.7.

Weitere Quellen: [100–106].

### 8.6. Psychiatrie

#### 8.6.1. Allgemeines

Psychiatrische Symptome und Krankheiten sind im Kontext von COVID‑19 in mehrfacher Hinsicht von Relevanz:

- Das Bedrohungsszenario der COVID‑19 Pandemie stellt eine psychische Belastung für die gesamte Bevölkerung dar und führte zu einer Zunahme von Symptomen von Angst, Depression und posttraumatischen Belastungsstörungen (PTSD).
- Patienten mit vorbestehenden psychischen Erkrankungen haben ein höheres Risiko an COVID‑19 zu erkranken und einen schwereren Verlauf zu entwickeln.
- Schwerere Verläufe von COVID‑19 können zu organischen psychischen Störungen wie Delirien führen
- Psychische Störungen sind auch Teil der Long COVID Symptomatik

#### 8.6.2. Psychiatrische Leitsymptome im Zusammenhang mit COVID-19

Die am häufigsten genannten psychiatrischen Symptome im Kontext von Long COVID sind Angst, depressive Verstimmungen, PTSD, postinfektiöse Müdigkeit, kognitive Störungen und Schlafstörungen. Darüber gibt aber noch viele andere Störungen, wie Zwangsstörungen, somatoforme Störungen, substanzbezogene Störungen, die gelegentlich auftreten oder sich gravierend verschlechtern können [107]. Die Häufigkeit psychischer Erkrankungen in einem Long COVID Sample wurde mit 39 % (nach 2 Monaten) angegeben [108].

##### Angst/Depression

Symptome von Angst und Depression werden ca. bei einem Viertel der Patient:innen mit Long COVID gefunden [109, 110].

Es ist empfehlenswert, Patient:innen mit somatischen Beschwerden von Long COVID diesbezüglich zu befragen. Umgekehrt ist es sinnvoll, Patienten, die sich primär mit psychischer Symptomatik präsentieren, nach dem Kontext mit COVID‑19 zu fragen.

Die Kardinalsymptome der Depression sind *gedrückte Stimmung, Freud- und Interesselosigkeit sowie Verminderung des Antriebs*. Angst tritt vorwiegend als generalisierte Angststörung (anhaltende, diffuse Angst, verschiedenste Lebensbereiche betreffend) und/oder in Form von Panikattacken (kurzdauernde Anfälle heftiger Angst ohne Anlass) auf.

Meist werden diese Störungen als reaktiv betrachtet [111–113]. Es gibt aber vereinzelt Hinweise darauf, dass die mit COVID‑19 verbundenen, inflammatorischen Prozesse das Risiko für Depressionen erhöhen können [114].

##### Posttraumatische Belastungsstörungen (PTBS)

Symptome von PTBS werden in der Akutphase der Pandemie sowohl bei Infizierten wie auch in der nicht-infizierten Allgemeinbevölkerung häufig gefunden und wurden auch im Kontext von Long COVID beschrieben [108, 115]. Kardinalsymptome der PTBS ist die ständige Wiederkehr der traumatischen Situation in Gedanken, Vorstellungen und Träumen und damit verbunden ein Rückzug aus dem Alltag.

##### Postinfektiöse Müdigkeit

(s. Abschn. 10.1./12.2., 12.4.2.)

##### Kognitive Störungen

(s. Abschn. 10.7./12.2., 13.3.)

##### Schlafstörungen

(s. Abschn. 10.10.)

**Weiters:** Zwangsstörungen, somatoforme Störungen, substanzbezogene Störungen [107]

Patient:innen mit somatischen Beschwerden von Long COVID sollten aktiv nach den genannten Symptomen gefragt werden!

#### 8.6.3. Methoden der psychiatrischen Abklärung

Untersuchende stehen vor der Aufgabe, psychischen Beschwerden den richtigen Stellenwert zuzuordnen. Zum einen geht es darum, psychische Beschwerden im Kontext von Long COVID nicht zu übersehen, zum anderen aber auch darum, somatische Beschwerden ohne fassbaren Befund nicht vorschnell als „psychisch“ abzustempeln.

Zum Screening nach Depression und Angst in der Praxis stehen zahlreiche kurze praktikable Fragebogen zur Verfügung, z. B. für Depression WHO‑5 [116], für Angststörungen GAD‑2 [117], für PTBS [118].

Derartige Fragebögen vermitteln keine Diagnose [119], bieten aber erste Anhaltspunkte für die weitere Exploration. Dabei muss man berücksichtigen, dass kürzere Fragebögen eine geringere Treffsicherheit haben als längere [120]. Basis der psychiatrischen Diagnostik ist noch immer das ärztliche Gespräch. Wichtig ist auch, ob es sich bei den psychischen Beschwerden um eine Erstmanifestation handelt, oder ob es bereits eine längere Vorgeschichte gibt. Weiters ist zu bedenken, dass psychische Beschwerden, die im Kontext von Long COVID auftreten, nicht zwangsläufig damit kausal zusammenhängen müssen, sondern auch andere Ursachen haben können.

#### 8.6.4. Differenzialdiagnosen

Differentialdiagnostisch sind bei Depression und Angst die möglichen (sehr seltenen) organischen Ursachen, wie endokrine Störungen und hirnorganische Veränderung zu bedenken.

##### Fatigue und kognitive Beeinträchtigungen

Neben den somatischen Ursachen für eine postinfektiöse Müdigkeit („Fatigue“) sind zentralnervöse Mechanismen zu diskutieren. Chronic Fatigue Syndrome/Myalgic Encephalitis (CFS/ME) ist eine in ihren Pathomechanismen unklare Erkrankung, wobei aber das Auftreten nach viralen Infektionen ein häufiges Merkmal ist [121]. Da es keine biologischen Marker gibt, beruhen die diagnostischen Kriterien auf Expertenkonsens. Allerdings wird die ICD-11 diagnostische Leitlinien für die Diagnose „postviral fatigue syndrome“ (PVFS) enthalten. Kognitive Beeinträchtigungen sind Teil dieses Syndroms. Wieweit die Fatigue-Symptome nach COVID‑19 zu dem meist chronisch verlaufenden CFS/ME [122] werden können, ist noch unklar [123].

Aus psychiatrischer Perspektive ist die wichtigste Differentialdiagnose die Depression, wobei das Hauptunterscheidungskriterium die fehlende traurige Verstimmung der Patienten mit PVFS ist, wobei es aber auch Überschneidungen bzw. Komorbiditäten geben kann.

### 8.7. Kinder

#### 8.7.1. Allgemeines

Im Gegensatz zu anderen Altersgruppen waren Kinder und Jugendliche bisher in der Pandemie in wesentlich geringerem Ausmaß von COVID‑19 direkt betroffen und sie zeigen zuallermeist einen sehr milden oder gar asymptomatischen Krankheitsverlauf [124, 125]. Allerdings haben Kinder und Jugendliche massiv unter den Folgen der Mitigationsmaßnahmen gelitten [126]. Besonders in dieser Altersgruppe stehen Symptome, welche nach einer SARS-CoV‑2 Infektion beobachtet werden, in manchen Fällen nicht in direktem Zusammenhang mit der Infektion selbst, sondern sind Folge der psychischen Belastung durch Mitigationsmaßnahmen gegen die Pandemie, ebenso wie bei Kindern und Jugendlichen ohne SARS-CoV‑2 Infektion.

Selten leiden jedoch auch Kinder gänzlich unabhängig von der Schwere des Krankheitsverlaufs, unter anhaltenden Beschwerden nach durchgemachter SARS-CoV‑2 Infektion [127, 128].

Prinzipiell zu unterscheiden gilt es zwischen Long COVID – ähnlich wie bei Erwachsenen – und dem Hyperinflammationssyndrom, welches auch MIS‑C (Multisystem Inflammatory Syndrome in Children) oder PIMS-TS (Paediatric Inflammatory Multisystem Syndrome temporarily associated with SARS-CoV‑2 infection) genannt wird. Dieses tritt bei Kindern, Jugendlichen und jungen Erwachsenen unter 21 Jahren etwa zwei bis acht Wochen nach einer SARS-CoV‑2 Infektion auf. ist jedoch eine eigene Krankheitsentität und zählt daher streng genommen nicht zu Long COVID. Dieses Krankheitsbild soll dennoch Eingang in dieses Papier finden, um Awareness zu schaffen, da es sich bei diesem Symptomkomplex um ein unter Umständen lebensbedrohliches Syndrom handelt (siehe unten).

#### 8.7.2. Long COVID

Long COVID bei Kindern bezieht sich auf die bei Erwachsenen geltenden Definitionen und beschriebenen, andauernden Symptome nach einer SARS-CoV‑2 Infektion. Diese Beschwerden umfassen ähnlich wie im Erwachsenenalter unter anderem Müdigkeit, Kopfschmerzen, Störungen von Geruchs- und Geschmackssinn, Kurzatmigkeit, Konzentrationsstörungen, mangelnde körperliche Belastbarkeit [127].

Bei der präliminären Zwischenauswertung einer Analyse von 755 Kindern im Alter von 0–14 Jahren mit SARS-CoV‑2 Infektion (durchgeführt von der MedUni Graz, AGES und ÖGKJ) zeigt sich, dass 11 % Beschwerden nach einem Monat und 6 % Beschwerden 3 Monate nach der SARS-CoV‑2 Infektion angeben, die mit Long COVID vereinbar sind, wobei ältere Kinder (10–14 Jahre) mit 16 % (Symptome nach > 1 Monat) und 10,7 % (Symptome nach > 3 Monaten) häufiger betroffen waren. Die in der Gesamtkohorte (alle Altersgruppen) am häufigsten angegebenen Symptome waren Müdigkeit (4,2 % nach 1 Monat bzw. 2,3 % nach 3 Monaten), Kopfschmerzen (2,6 % bzw. 1,7 %), Kurzatmigkeit (2,1 % bzw. 1,3 %), Konzentrationsstörungen (1,9 % bzw. 1,1 %), eingeschränkte körperliche Belastbarkeit (2,1 % bzw. 1,1 %), gefolgt von Husten, Halsschmerzen, Gelenks‑/Gliederschmerzen und Bauchschmerzen (nach 1 Monat jeweils 0,7 bis 1,1 % bzw. nach 3 Monaten 0,4 % bis 0,7 %). Störungen von Geruchs- und Geschmackssinn wurden bei Kindern unter 10 Jahren seltener als 1 % angegeben, bei Kindern > 10a jedoch in 5,6 % (nach 1 Monat) bzw. 3,3 % (nach 3 Monaten) berichtet. Diese Symptome wurden von den Familien als mit der Infektion im Zusammenhang stehend beurteilt. Eine Abgrenzung gegenüber Beschwerden anderer Ursache, z. B. durch Isolation im Rahmen der Pandemie-Maßnahmen, ist jedoch im Einzelfall schwierig, sodass die Rate der tatsächlichen Long COVID Symptome unter den angegebenen Häufigkeiten liegen dürfte. Eine kürzlich veröffentlichte Arbeit mit Daten von 1734 SARS CoV‑2 positiven Kindern und Jugendlichen (5–17 Jahre) aus dem Vereinigten Königreich zeigte nach COVID‑19 anhaltende Symptome nach 1 Monat bei 4,4 % (77 von 1734) und nach 3 Monaten bei 1,8 % (25 von 1379). In dieser Arbeit waren höheres Alter der Kinder sowie ein schwererer Krankheitsverlauf mit anhaltenden Beschwerden positiv korreliert. Auch in dieser Arbeit erwiesen sich Müdigkeit und Kopfschmerzen als die am häufigsten angegebenen Symptome [129].

Die diagnostische Abklärung von Long COVID im Kindesalter sollte sich wie bei Erwachsenen an den beschriebenen Beschwerden und dem Ausschluss anderer Differenzialdiagnosen orientieren (s. dazu Kap. 8 und 9). Die diagnostische Abklärung kann sich dabei an den einzelnen Kapiteln dieser Leitlinie orientieren, wobei auf die pädiatrischen Gegebenheiten Rücksicht genommen werden muss. Neben der Abklärung anderer organischer Differentialdiagnosen ist eine Berücksichtigung psychischer Ursachen von essenzieller Bedeutung.

Neben einer Basisblutabnahme (mit Blutbild und Chemie) sollten je nach Beschwerdebild EKG, Blutdruckmessungen, Lungenfunktion und gegebenenfalls 24 h Blutdruck, Belastungsergometrie und Herzultraschall in Betracht gezogen werden. Auch eine klinisch psychologische Evaluierung zu diagnostischen Zwecken oder auch zur Entlastung bei über viele Wochen bestehenden Symptomen sollte erwogen werden.

#### 8.7.3. MIS-C/PIMS-TS

Das klinische Erscheinungsbild variiert und reicht von Fieber (mit oder ohne Bauchschmerzen) ohne andere erklärbare Ursache mit stark ausgelenkten Entzündungsparametern bis zu einem Kawasaki Syndrom ähnlichem Phänotyp oder einer Präsentation mit Schock, Gerinnungsstörung und Multiorganversagen. Prinzipiell handelt es sich um ein progressives Erscheinungsbild, das zumeist mild beginnt und innerhalb einiger Tage zu einer schweren Dysfunktion mehrerer Organsysteme führen kann [130, 131].

Ähnlich wie bei Kawasaki Syndrom bilden sich bei einem Teil der Kinder Koronaraneurysmen.

Abgesehen von der relativ späten Symptomentwicklung nach einer Infektion unterscheidet sich Mis‑C auch bezüglich der auffindbaren Biomarker und Zytokine von der Hyperinflammation bei COVID‑19 im Erwachsenenalter [132].

Die WHO definiert MIS‑C wie folgt:

- Kinder und Jugendliche ≤ 19 Jahre mit Fieber ≥ 3 Tage
- UND zwei der folgenden Kriterien:
  - Exanthem oder bilaterale non-purulente Konjunktivitis oder mukokutane Entzündungszeichen (Mund, Hände, Füße).
  - Hypotension oder Schock.
  - Zeichen einer myokardialen Dysfunktion, Perikarditis, Valvulitis oder Koronaranomalien.
  - Zeichen einer Gerinnungsstörung (PT, PTT, erhöhtes D‑Dimer)
  - Akute gastrointestinale Beschwerden (Diarrhoe, Erbrechen oder Abdominalgie)
- UND erhöhte Entzündungsparameter (Blutsenkung, C‑reaktives Protein oder Procalcitonin)
- UND keine andere offensichtliche Ursache der Entzündung, wie bakterielle Sepsis, Toxisches Schocksyndrom
- UND Evidenz für COVID‑19 (positiver PCR-, Antigentest oder Serologie) oder wahrscheinlicher Kontakt zu SARS-CoV‑2.

(adaptiert nach WHO [133], Mai 2020)

Die Inzidenz ist derzeit ebenfalls nicht ausreichend geklärt. In einer Metaanalyse, im Zuge derer Daten aus 26 Ländern und insgesamt 7780 Kinder mit SARS-CoV‑2 Infektion eingeschlossen wurden, wurde die Inzidenz auf 0,14 % aller SARS-CoV‑2 Infektionen im Kindes- und Jugendalters geschätzt [134]. Probleme bei der Inzidenzabschätzung sind das Fehlen eines allgemein gültigen Meldesystems für MIS‑C, die vielfältige klinische Präsentation und dass die Zahl SARS-CoV‑2 infizierter Kinder vermutlich höher ist als die tatsächlich gemeldeten Fälle. Von Februar 2020 bis Jänner 2021 wurden in Österreich 51 Fälle eines Mis‑C gemeldet. Zwanzig davon benötigten die Aufnahme auf einer Intensivstation. Ein Jugendlicher benötigte eine extracorporale Membran-Oxygenierung (ECMO). Im selben Zeitraum sind 50.378 bestätigte SARS-CoV‑2 Infektionen in dieser Altersgruppe registriert worden, was einer Inzidenz von 0,1 % aller SARS-CoV‑2 Infektionen im Kindes- und Jugendalters entsprechen würde (ÖGKJ, AGES 2021).

Der Pathomechanismus ist bisher unbekannt. Es dürfte sich jedoch um ein multifaktorielles, immunologisches Geschehen nach Kontakt des Körpers mit dem Virus handeln.

Die Überlappungen mit dem Kawasaki Syndrom deuten auf eine Vaskulitis und ein autoimmunologisches Geschehen hin. Diskutiert werden unter anderem eine Kombination aus einer überschießenden T‑Zell Antwort und dem Auftreten von Autoantikörpern nach einer SARS-CoV‑2 Infektion [132].

In der Therapie kommen vornehmlich intravenöses Immunglobulin und hochdosierte Glukokortikoide zum Einsatz. Ein hochfieberhaftes Zustandsbild bei Kindern- und Jugendlichen wenige Wochen nach (möglicher) SARS-CoV‑2 Infektion ohne eindeutige andere Ursache sollte an dieses Krankheitsbild denken lassen. Eine frühzeitige Kontaktaufnahme mit definierten Kompetenzzentren und ein interdisziplinäres Management sind essenziell [135, 136].

### 8.8. Zusammenfassung

Long COVID kann sich in Symptomen aus einer Vielzahl von Organsystemen manifestieren. Psychische Komorbiditäten bzw. Begleiterscheinungen oder Folgen sind häufig, zu beachten sind auch soziale Kofaktoren. Unterschiedliche Ätiologien der Symptome sind zu differenzieren: organische Folgen der Akuterkrankung, nicht vollständig geklärte funktionelle Störungen, Aggravierung vorbestehender Erkrankungen, Anpassungs- und/oder Somatisierungstörungen.

**Figure 3:**
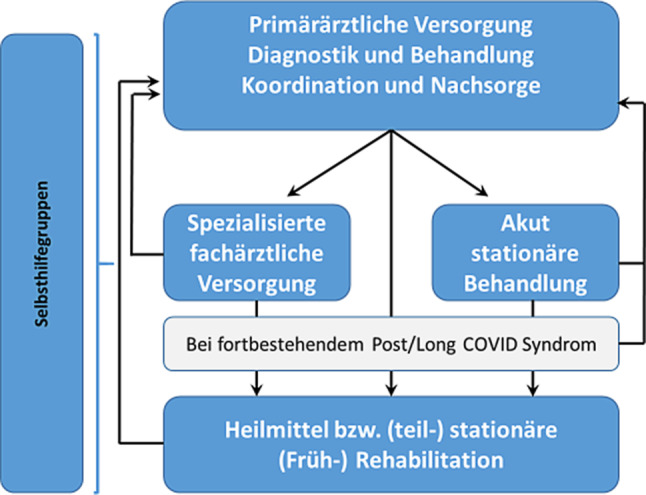

Abb. 3 zeigt einen Vorschlag für die sinnvolle Versorgungsorganisation.

#### Empfehlung

Long COVID verlangt nach einer ganzheitlich orientierten und personenzentrierten Vorgangsweise. Erstanlaufstelle für die Einordnung und Abklärung von Symptomen, die mit einer vorangegangenen Erkrankung durch SARS.CoV‑2 in Zusammenhang stehen könnten, ist sinnvollerweise die hausärztliche Primärversorgung, die aufgrund ihrer Fachdefinition (Degam, Wonca) am besten geeignet ist: sowohl für die Abklärung, als auch für Behandlungsplanung bzw. die gezielte Weiterleitung an geeignete Kooperationspartner (Sonderfächer, Medizinberufe, psychologische und soziale Beratungsstellen).

## 9. Die häufigsten Symptome – Differenzialdiagnostik

Verdachtsbegründend ist jedes der in dieser Leitlinie beschriebenen Symptome (s. Kap. 6), sofern es in zeitlich passendem Abstand von einer Infektion mit SARS-CoV‑2 auftritt. Zudem ist aufgrund des neuen Krankheitsbildes damit zu rechnen, dass Symptome und Befunde bisher noch unberichtet geblieben sind. Offene Aufmerksamkeit gegenüber Symptomen wird empfohlen, die anderweitig nicht erklärt sind und in passendem Zusammenhang auftreten.

Unerklärte, persistierende oder ausgeprägte Symptome vor allem aus dem beschriebenen Spektrum, ohne bekannte abgelaufene Erkrankung an COVID‑19 sollten an eine okkulte Infektion mit SARS-CoV‑2 denken lassen, wenn keine andere Ursache identifizierbar ist [8].

### 9.2. Grundlegendes zum diagnostischen Ablauf

Jedes der Symptome kann trotz der zeitlichen Assoziation unabhängig von COVID‑19 aufgetreten sein.

Der erste diagnostische Schritt ist, wie immer, die sorgfältige **fokussierte Anamnese**, mit einigen Besonderheiten:

- Gegenwärtige Beschwerden und Symptome – exakte Exploration
  - Beginn mit offenen Fragen: welche Symptome bemerken Sie?
  - Präzisierung mittels konkreter Nachfrage entsprechend der angegebenen Symptome
  - Aktives Fragen nach weiteren, nicht erwähnten Wahrnehmungen ist in Zusammenhang mit Long COVID besonders wichtig
- Vorbestehende Erkrankungen und Medikationen
- Infektionsvorgeschichte und -verlauf, insbesondere:
  - Gab es Hinweise auf eine kardiale Beteiligung während der akuten Erkrankungsphase?
  - Gab es Hinweise auf eine PAE/thromboembolisches Geschehen (CT, D‑Dimer)? Erfolgte eine andere Bildgebung der Lunge?
  - Gab es Zeiten mit Atemnot, erheblicher Schwäche, Sauerstoffbedarf (Zeitraum und Menge erheben)
  - Gab es neurologische Symptome?
  - Andere Komplikationen?
  - Behandlung: was ist zu welchem Zeitpunkt geschehen?

Danach erfolgt die fokussierte **klinische Untersuchung** entsprechend der von den Patient:innen berichteten Situationen.

Aufgrund der Zusammenschau der Ergebnisse wird über die folgenden nötigen **Basisuntersuchungen** (Labor, apparativ etc.) und eine ev. nötige **Weiterleitung** in den spezialisierten Bereich entschieden.

### 9.3. Diagnostische Zielsetzungen

#### 1: Ausschluss potenziell gefährlicher Verläufe – „Red flags“

Wie immer, wenn sich eine Patient:in mit einem mehrdeutigen Symptom vorstellt, erfolgt zunächst die **Beurteilung der Dringlichkeit**. Mittels kurzer, fokussierter Anamnese und darauffolgendem **zielgerichteten** klinischen Assessment wird ein potenziell gefährlicher Verlauf ausgeschlossen. Die Alarmsignale sind abhängig vom jeweils präsentierten Symptom und werden nach den gleichen Prinzipien beurteilt, wie in allen anderen Situationen (Vitalzeichen, Allgemeinzustand, Dynamik der Beschwerden).

Spezifische red flags in Zusammenhang mit COVID‑19 z. B.:

- Hinweise auf akute respiratorische Insuffizienz: Ruhedyspnoe – Hypoxämie in Ruhe oder bei Belastung
- Hämoptysen
- Vermutet kardiogener Thoraxschmerz
- Hinweise für ausgeprägte Kreislaufinstabilität
- bei Kindern und Jugendlichen: Hinweise auf Multisystem Inflammatory Syndrome
  - (persistierendes) Fieber auftretend 2–8 Wochen nach Sars-Cov‑2 Infektion (s. a. Abschn. 8.7.3.)

#### 2: Abgrenzung fassbarer Pathologien (mit ev. behandelbarer Grundkrankheit)

Ein zeitlicher Zusammenhang muss kein kausaler Zusammenhang sein. Es muss nach COVID‑19 mit allen Störungen gerechnet werden, die auch sonst möglich sind. Diese sind daher so wie üblich leitliniengerecht abzuklären bzw. auszuschließen. Zusätzlich ist auf spezielle Pathologien besonderes Augenmerk zu legen, die in besonderem Maße im Gefolge von COVID‑19 auftreten, wie in Kap. 8 beschrieben.

#### 3: Evaluierung funktioneller, COVID-19 assoziierter Folgeerscheinungen mit Einstufung des Handlungsbedarfs

Erhebung aller zum Untersuchungszeitpunkt wahrgenommenen Symptome (offene Fragen).

**Figure 4:**
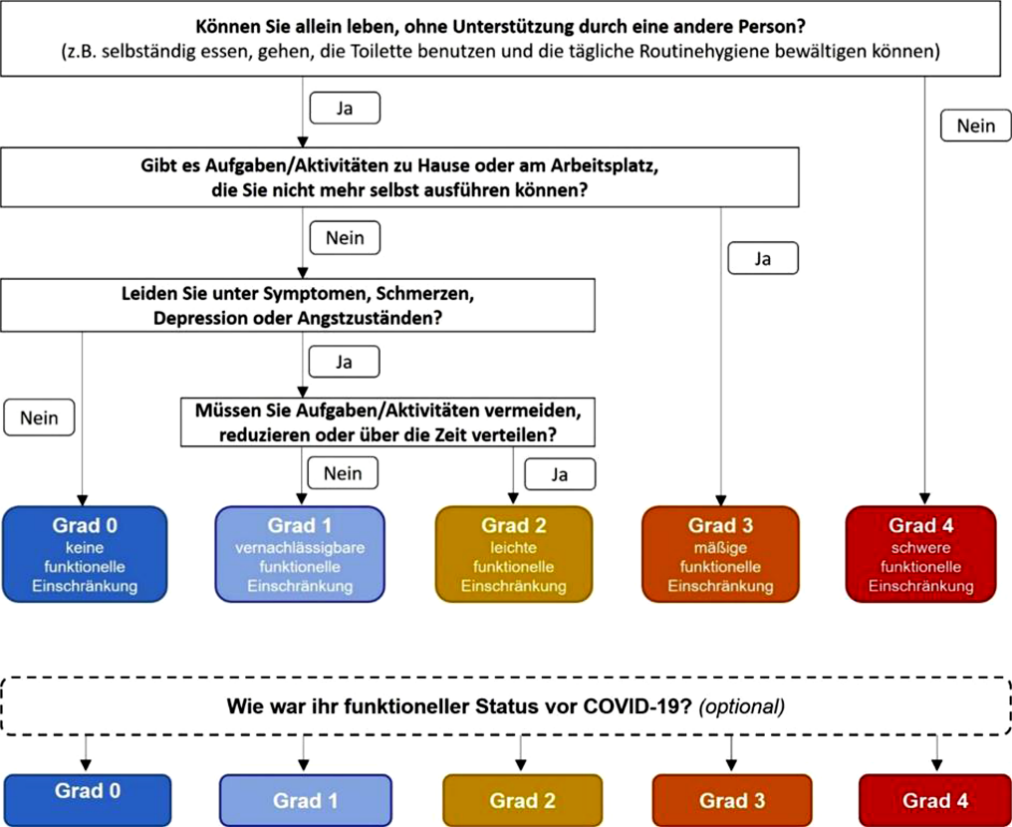

Bewertung des subjektiven Leidensdrucks – möglichst mittels geeigneter Skalen – s. Abb. 4 (Post-Covid-19 Skala des funktionellen Status), Tab. 1: Borgskala, Tab. 2: Fatigue Assessment Scale (FAS), Tab. 3: mMRC-Skala.

**Table 1:** 

Anstrengung	Borgskala	Luft
Einschlafen	0	Überhaupt keine Atemnot
Überhaupt keine Anstrengung	1	Gerade wahrnehmbare
Sehr, sehr leicht	2	Sehr milde Atemnot
Sehr leicht	3	Milde Atemnot
Leicht	4	Mäßige Atemnot
Moderat gemütlich	5	Mittelschwere Atemnot
Anstrengend	6	Schwere Atemnot
Hart	7	Sehr schwere Atemnot
Sehr hart	8	Sehr, sehr schwere Atemnot
Sehr, sehr hart	9	Fast maximale Atemnot
Maximale Anstrengung	10	Maximale Atemnot

**Table 2:** 

Die folgenden zehn Aussagen betreffen Ihr normales Befinden. Bitte umkreisen Sie die Antwort, die am besten zu Ihnen passt. Beantworten Sie bitte jede Frage, auch wenn Sie momentan keine Beschwerden haben. Sie können pro Aussage zwischen 5 Antwortmöglichkeiten wählen, variierend von „niemals“ bis „immer“. 1. **Niemals** 2. **Manchmal** (d. h. monatlich oder weniger) 3. **Regelmäßig** (d. h. ein paar Mal pro Monat) 4. **Oft** (d. h. wöchentlich) 5. **Immer** (d. h. täglich)					
	**Niemals**	**Manchmal**	**Regelmäßig**	**Oft**	**Immer**
1. Ich leide unter Ermüdungserscheinungen.	O	O	O	O	O
2. Ich bin schnell müde.	O	O	O	O	O
3. Ich finde, dass ich an einem Tag wenig mache.	O	O	O	O	O
4. Ich habe genug Energie für den Alltag.	O	O	O	O	O
5. Körperlich fühle ich mich erschöpft.	O	O	O	O	O
6. Es fällt mir schwer Sachen anzufangen.	O	O	O	O	O
7. Es fällt mir schwer klar zu denken.	O	O	O	O	O
8. Ich habe keine Lust etwas zu unternehmen.	O	O	O	O	O
9. Ich fühle ich geistig erschöpft.	O	O	O	O	O
10. Wenn ich mit etwas beschäftigt bin, kann ich mich gut darauf konzentrieren.	O	O	O	O	O

Anmerkung: die Punkte 4 und 10 sind invers zu werten!

**Table 3:** 

Atemnot	Punkte
Nie Atemnot, ausser bei maximaler körperlicher Anstrengung	0
Atemnot bei Anstrengung in der Ebene oder leichter Steigung	1
Atemnot bei normalem Gehtempo (altersentspr.) oder häufigere Atempausen	2
Atemnot nach 100 m in der Ebene oder nach wenigen Minuten	3
Atemnot beim Anziehen, Patient kann das Haus nicht verlassen	4

### Beurteilung der Funktionseinschränkung bei Long COVID

Diese erfolgt im Gespräch mit der Patient:in und umfasst die Erhebung aller zum Untersuchungszeitpunkt wahrgenommenen Symptome (offene Fragen). Eine Objektivierung, auch zur vergleichenden Verlaufsbeobachtung, wird durch die Verwendung der Post-COVID-19 Skala des funktionellen Status erleichtert (s. Abb. 4).

Die Skala wurde von Klok et al. aus einer für andere Situationen bereits bewährten Skala weiterentwickelt und für die Einschätzung des funktionellen Status bei Personen nach COVID‑19 mit einem zumindest 12 Wochen zurückliegenden Symptombeginn validiert. Sie dient auch zur Verlaufsbeobachtung [137].

#### Empfehlung

Nicht jedes Symptom erfordert die sofortige umfassende Abklärung. Nach Ausschluss eines potenziell gefährlichen Verlaufs und nach entsprechender Basisuntersuchung mit Ausschluss eines fassbaren organischen Substrats kann bei milder Symptomatik in vielen Fällen abwartend beobachtet werden. Die Quote der Spontanheilungen dürfte hoch sein.

## 10. Zuordnung der häufigsten Symptombilder

Die Symptombilder in diesem Kap. gehören zu den häufigsten, die nach COVID‑19 beschrieben werden. Sie sind überwiegend mehrdeutig – sie können also mit einer vorangegangenen SARS-CoV‑2 Infektion in einem kausalen Zusammenhang stehen – ein solcher ergibt sich aus der zeitlichen Assoziation jedoch nicht zwingend.

Beurteilungsgrundlagen für weiterführende Diagnostik bzw. Therapiebedarf

- Ausmaß der gesundheitlichen Einschränkung
- Dynamik der Symptomatik, Dauer
- Körperliche, kognitive und psychische Leistungsfähigkeit
- Individuelle Situation und Prioritäten der Betroffenen

### 10.1. Postinfektiöse Müdigkeit/Abgeschlagenheit („Fatigue“)

#### 10.1.1. Allgemeines

Auch nach anderen Viruserkrankungen (u. a. Influenza, EBV, CMV, Enteroviren, HHV6, Masern) werden z. T. länger anhaltende Krankheitssymptome beobachtet. Daher lässt sich insbesondere das oft vorherrschende Symptom der ausgeprägten postviralen Erschöpfung und Müdigkeit (auch als Fatigue bezeichnet) ggf. auch dieser Entität der postinfektiösen Müdigkeit zuordnen.

Hinweisend können folgende Symptome sein, die meist in Kombination auftreten [10, 50]:

- Eine im Vergleich zur Zeit vor der Erkrankung substanzielle Einschränkung der Fähigkeit zu beruflichen, schulischen, sozialen oder persönlichen Aktivitäten mit plötzlichem oder definiertem Beginn
- Abgeschlagenheit nach körperlicher Belastung (engl. post-exertional malaise)
- Nicht erholsamer Schlaf
- Kognitive Einschränkungen
- Orthostatische Intoleranz – POTS (Posturales Tachykardie-Syndrom)
- Die Erschöpfung hängt nicht mit einer organischen Erkrankung oder anhaltender Anspannung zusammen

##### Empfehlung

Bei geringer bis mäßiger Beeinträchtigung aufgrund postinfektiöser Müdigkeit und Fehlen von red flags sollte eine weiterführende Diagnostik nur bei anhaltenden Beschwerden über 12 Wochen angestrebt werden. Eine Überdiagnostik ist zu vermeiden, um eine iatrogene Fixierung hintanzuhalten.

#### 10.1.2. Ausschluss potenziell gefährlicher Verlauf (sofortiger Handlungsbedarf)

Erfassung der subjektiven und objektiven Beeinträchtigung und objektive Bewertung der akuten Gefährdung (u. a. Symptome ggf. im Zusammenhang mit Depression, Herzinsuffizienz, Tumorerkrankungen, schweren Anämien, Stoffwechselerkrankungen, Intoxikation).

#### 10.1.3. Zuordnung

##### Anamnese

Ausgeprägte/ungewöhnliche Belastungsintoleranz (post exertional malaise/PEM), Vorliegen und/oder Verschlechterung anderer chronische Erkrankungen, psychosoziale Umstände, persönliches Verhalten, Risikoverhalten, Schlafqualität, Schnarchen, Körpergewicht, Vorstellungen der Patientin/des Patienten zur Ätiologie des Symptoms, (Dauer‑) Medikamente.

- Die ausgeprägte Müdigkeit nach COVID‑19 erlaubt es oft nicht, Alltagsaktivitäten oder dem Beruf nachzugehen, meist auch in Zusammenhang mit Konzentrationsstörungen
- **Abgrenzung zu anderen Krankheiten**: Depressio; Schlafapnoe/Narkolepsie; neuromuskuläre Erkrankungen u. a. (s. unten)
- Die Evaluierung kann durch die Fatigue Assessement Scale (s. Tab. 2) unterstützt werden. Andere mögliche Instrumente zur Objektivierung: FAS SFQ, Rand-36 (energy/fatigue) [57].

##### Klinische Untersuchung

- Körperliche Untersuchung (Abdomen, Lymphregionen, Herz, Puls und Blutdruck, Schleimhäute, Atemwege, Muskeltrophik, -kraft, -tonus, -eigenreflexe)

##### Basisdiagnostik

- RR, po2, ggf. EKG, BZ, BB, CRP

##### Weitere Untersuchungen

- **Nur** bei gegebenem Anlass: Belastungstest, kardiologische Untersuchung (Ergo, Echo), neurologische Begutachtung, ggf. nach Vorbefunden weitere Laborparameter: Elektrolyte, Kreatinin, GFR, Transaminasen, Glucose, TSH, Ferritin, HIV, NNR-Funktion, Immunglobuline

#### 10.1.4. Procedere

##### Abwartendes Offenhalten nach Ausschluss eines potenziell gefährlichen Verlaufs

- Patient:innen ausreichend Erholung ermöglichen (ggf. durch Krankenstandsmeldung)
- Nach viralen Erkrankungen sind postvirale Erschöpfungszustände im Rahmen der Rekonvaleszenz häufig und vor allem häufig selbstlimitierend
- Patient:innen über die zu erwartende Selbstlimitierung der Symptome aufklären

##### Fachspezifische Abklärung

Kein generelles Screening auf mögliche Ursachen, sondern nur Abklärung von Krankheitsbildern, bei denen es objektivierbare Anhaltspunkte gibt.

##### Selbstmonitoring und -management

(Pulsmesser, Symptomtagebücher), um individuelle Belastungsgrenze zu erkennen [138].

#### 10.1.5. Weitere wichtige Differenzialdiagnosen

- Kardiale oder pulmonale Erkrankungen
- Anämie
- Schilddrüsenfunktionsstörungen
- Andere hormonelle Imbalancen
- Chronische Infektionen anderer Genese
- Stoffwechselerkrankungen (Diabetes!)
- Muskuläre Schwäche
- Überlastungssituationen
- Psychische Erkrankungen
- Gestörter Schlaf

**Behandlung** s. Abschn. 12.4.2.

##### Empfehlung

Bei geringer bis mäßiger Beeinträchtigung aufgrund postinfektiöser Müdigkeit und Fehlen von red flags sollte eine weiterführende Diagnostik nur bei anhaltenden Beschwerden über 12 Wochen angestrebt werden. Eine Überdiagnostik ist zu vermeiden, um eine iatrogene Fixierung hintanzuhalten.

### 10.2. Schwäche/Leistungsminderung

#### 10.2.1. Allgemeines

Die Ausprägung der Leistungseinschränkung nach akuter Infektion ist wesentlich abhängig vom Verlauf der Erkrankung [139]. Drei bis sechs Monate nach Hospitalisierung konnte in einer kleinen Pilotstudie eine maximale Wattleistung von 92 % aufgezeigt werden mit eingeschränkter maximaler Sauerstoffaufnahme von 73 % der Norm [140]. Auffallend dabei war eine deutlich erhöhte Atemarbeit bei Belastung mit Hypokapnie auch bei Patient:innen mit milden Verläufen und ohne residualen Veränderungen im Thorax-CT, sodass doch die muskuläre Einschränkung sowohl der peripheren Muskulatur als auch der Atemmuskulatur ursächlich zu sein scheint. Eine begleitende Stress-Echokardiographie ergab keinen Hinweis für eine pulmonale Hypertonie bei Belastung.

#### 10.2.2. Ausschluss potenziell gefährlicher Verlauf

- Erfassung der subjektiven und objektiven Beeinträchtigung (Pulsoxymetrie, Belastungstests)
- Weitere „red flags“: Vitalzeichen (!), rezentes Auftreten; plötzliche oder rasche Zunahme, zusätzliche Symptome (wie Stauungszeichen, Hautkolorit, kognitive Veränderungen, starkes Durstgefühl)

#### 10.2.3. Zuordnung

**Anamnese** (Zeitpunkt des Auftretens, Dynamik, Belastungsabhängigkeit):

- Einschätzung einer möglichen depressiven Symptomatik
- Atemnot
- Gewichtsab-/-zunahme, Ödeme
- Blutungszeichen
- Fieber, Nachtschweiß
- Schmerzen (dauerhaft/nur bei Belastung)
- Schlafanamnese

##### Klinische Untersuchung

- fokussierter Status: Auskultation Herz und Lunge, Palpation, Suche nach Stauungszeichen, Aszites, Hautkolorit

##### Basisdiagnostik

- SpO2, RR (bei entsprechenden Hinweisen im Liegen und Stehen)
- BB, BSG/CRP, BZ, Kreatinin, GPT, GGT, Natrium, Kalium, Calcium, TSH, Harn
- Belastungstests
- Einschätzung des Schweregrads s. Abb. 4 (Skala funktionelle Beurteilung)

#### 10.2.4. Procedere

**Abwartendes Offenhalten; **Wenn eine erste Abklärung inkl. Labor keine Hinweise auf einen abwendbar gefährlichen Verlauf zeigt und keine weiteren anamnestischen und klinischen Anzeichen für strukturelle Erkrankungen gefunden werden, kann in Absprache mit der/dem Betroffenen mit einer weiterführenden Abklärung zugewartet werden (Fehlen von red flags).

- Zeichen einer begleitenden/ursächlichen Dyspnoe. Abklärung s. Abschn. 9.4.
- Protrahierte Leistungsminderungen sind auch nach anderen Virusinfekten bekannt s. Abschn. 9.1.
- Klinische Kontrolle bei der Hausärztin/dem Hausarzt je nach Situation, zumindest nach 4–6 Wochen [141]. Auf die Notwendigkeit der Wiedervorstellung bei Verschlechterung ist dokumentiert hinzuweisen!

##### Fachspezifische Abklärung

- Hinweise auf kardiale Ursachen: Abklärung nach etablierten Routinen: Herzinsuffizienz, KHK, pulmonal-arterielle Hypertension, Arrhythmien. Bei Z. n. COVID‑19 ist u. a. an entzündliche oder postentzündliche Ätiologien zu denken (s. Kap. 8)
- Hinweise auf pulmonale Ursachen: Abklärung nach etablierten Routinen.

#### 10.2.5. Weitere wichtige Differenzialdiagnosen

- Muskuläre Dekonditionierung z. B. nach einer protrahierten Erkrankung, Anämie, Adipositas, Skeletterkrankungen, neuromuskuläre Erkrankungen
- Es ist wie immer an die Möglichkeit des Vorliegens anderer Erkrankungen mit ausschließlich zeitlicher Koinzidenz zu denken.

**Behandlung** s. Abschn. 12.2., 12.3., 12.4.2.

### 10.3. Riech- und Schmeckstörungen

#### 10.3.1. Allgemeines

Einer Riechstörung können zwei pathophysiologische Mechanismen zugrunde liegen: Blockade des Duftstoff-Transports zur Riechschleimhaut im Nasendach oder eine sensori-neurale Schädigung (Riechnervenzellen oder zentrale Strukturen wie Bulbus olfactorius oder Hirnareale).

Die COVID-bedingte Riechstörung fällt in die zweite Gruppe, in die auch vor Beginn der COVID‑19-Pandemie andere Viren (z. B. Influenza‑, Parainfluenza‑, Rhinoviren) unter dem Begriff der post-infektiösen bzw. post-viralen Riechstörung zusammengefasst wurden [87].

#### 10.3.2. Ausschluss potenziell gefährlicher Verlauf

Einige Hirntumore können mit Riechverlust einhergehen (Meningeome, Tumore der vorderen Schädelgrube), auch Insulte. Dabei stellt der Riechverlust normalerweise kein Einzelsymptom dar.

#### 10.3.3. Zuordnung

##### Anamnese

- Zeitpunkt des Auftretens, Verlauf, genaue Symptomatik erfragen: Hyposmie, Dysosmie, Trauma, Chemo- oder Strahlentherapie, Operation? Weitere Symptome (neurologisch oder internistisch)?

##### Klinische Untersuchung

- Hinweise auf sinu-nasale Erkrankungen? Gezielte neurologische Untersuchung

#### 10.3.4. Procedere

Wenn es einen klaren zeitlichen Zusammenhang mit der SARS-CoV‑2 Infektion gibt, und Hinweise auf andere Erkrankungen fehlen, kann ohne weiterführende Untersuchung von einer COVID‑19 assoziierten Riechstörung ausgegangen werden.

#### 10.3.5. Weitere wichtige Differenzialdiagnosen

- Sinunasale Erkrankungen (z. B. chron. Sinusitis, Allergie, Septumdeviation, Rhinopathia gravidarum, Tumoren)
- Schädel-Hirn-Trauma
- Andere Infekte (nasal oder systemisch, z. B. Influenza, common-cold)
- Zentrale Ursachen (z. B. Meningeom, Insult)
- Neurodegenerative Erkrankungen (z. B. M. Parkinson, M. Alzheimer)
- Internistische Erkrankungen (z. B. Leber‑, Nieren‑, Schilddrüsenerkrankungen)
- Medikamentös-toxische Einflüsse (z. B. Chemo‑, Strahlentherapie)
- Iatrogen (Neurochirurgische OP, Nasen/NNH-OPs)
- Angeborene Riechstörung (z. B. Kallmann-Syndrom)
- Idiopathische Riechstörung (Ausschlussdiagnose!)

**Behandlung **s. Abschn. 12.4.4.

##### Riechtraining

Abb. 2: Riechtraining: Patient:inneninformation zur Gestaltung des Riechtrainings

### 10.4. Dyspnoe

#### 10.4.1. Ausschluss potenziell gefährlicher Verlauf

- Erfassung der subjektiven und objektiven Beeinträchtigung (Pulsoxymetrie, Belastungstests). Bei subjektiver ausgeprägter Atemnot und/oder SpO2-Werten < 93 % (bei vorbestehender chron. respiratorischer Erkrankung: deutlicher Abfall) besteht akuter Handlungsbedarf [9].
- Weitere „red flags“: rezentes (neues) Auftreten; plötzliche oder rasche Zunahme, zusätzliche Symptome (Thoraxschmerz, Husten, Hämoptysen, Fieber, Stauungszeichen, Hautkolorit, EKG-Veränderungen, kognitive Veränderungen)

#### 10.4.2. Zuordnung

##### Anamnese (Zeitpunkt des Auftretens, Dynamik, Belastungsabhängigkeit):

- Tatsächlich Atemnot – oder mangelnde Leistungsfähigkeit, Müdigkeit bis Fatigue? Belastungsintoleranz ohne objektivierbare Dyspnoe?
- Dyspnoe im Rahmen von Long COVID äußert sich vor allem als Kurzatmigkeit bei Belastung, und findet sich häufiger nach schwerem Verlauf, aber auch nach nicht-hospitalisiertem Verlauf (in ca. 10 %) [142]
- Frage nach Vorerkrankungen und weiteren Symptomen
- Einstufung Dyspnoe-Ausmaß: nach NYHA-Klassifikation, Borg-Skala (Tab. 1), mMRC (Tab. 3)

##### Klinische Untersuchung

- fokussierte Basisuntersuchung: Auskultation Herz und Lunge, Suche nach Stauungszeichen, Hautkolorit, Beurteilung der Atmung mit Objektivierung der Dyspnoe: Atemfrequenz, Sprechen, Belastungstests unter Beobachtung (Gehtest, Stiegensteigen) und Einstufung nach NYHA, Abgrenzung musk. Schwäche (s. Tab. 3: *mMRC-Skala*)

##### Basisdiagnostik

- Jedenfalls: SpO2 (auch im Selbstmonitoring!), RR, KBB, CRP

##### Weitere Untersuchungen

- weitere Basisdiagnostik: Bei klinisch objektivierbarer Dyspnoe: Thoraxröntgen, EKG, NTproBNP. Fehlende Hinweise auf kardiale Genese: Spirometrie, ev. D‑Dimer bei entsprechenden Verdachtsmomenten.

#### 10.4.3. Procedere

##### Abwartendes Offenhalten

Wenn eine erste Abklärung keine Hinweise auf reduzierte Leistungsfähigkeit oder sonstige deutlich Beeinträchtigungen erbringt, und keine weiteren anamnestischen und klinischen Anzeichen für strukturelle Erkrankungen gefunden werden, kann in Absprache mit der/dem Betroffenen mit einer weiterführenden Abklärung zugewartet werden (Fehlen von red flags).

- Bis zu ca. 6 Wochen nach ambulanter Erkrankung ist eine milde Kurzatmigkeit häufig. Sollte diese anamnestisch bereits gebessert und mild sein, sind bei unauffälligem klinischem Status, unauffälligem Routinelabor und unauffälligem Thorax-Röntgen vorerst keine weiterführenden Untersuchungen erforderlich.
- Klinische Kontrolle beim Hausarzt je nach Situation, zumindest nach 4–6 Wochen [141].
  Auf die Notwendigkeit der Wiedervorstellung bei Verschlechterung ist dokumentiert hinzuweisen!

##### Fachspezifische Abklärung

- Hinweise auf kardiale Ursachen: Abklärung nach etablierten Routinen.
  Herzinsuffizienz, KHK, pulmonal-arterielle Hypertension, Arrhythmien. Bei Z.n.COVID‑19 ist z. B. auch. an entzündliche oder postentzündliche Ätiologien zu denken s. Kap. 7
- Hinweise auf pulmonale Ursachen: Abklärung nach etablierten Routinen.
  Z.n.COVID‑19: bei Risikofaktoren oder klinischen Hinweisen (akutes Einsetzen, akute Verschlechterung) Ausschluss einer PE (D-Dimer).
  - Bei pathologischem Thorax-Röntgen sowie ausgeprägter Dyspnoe mit Beeinträchtigung der Leistungsfähigkeit oder bei Verschlechterungstendenz, ist die fachärztliche Abklärung erforderlich, um Lungenschäden nach COVID‑19 zu definieren, wie residuale Pneumonie, organisierende Pneumonie, Hinweise für Fibrose, chronische PE – s. Abschn. 8.1.

#### 10.4.4. Weitere wichtige Differenzialdiagnosen

Muskuläre Dekonditionierung z. B. nach einer protrahierten Erkrankung, Anämie, Adipositas, Skeletterkrankungen, neuromuskuläre Erkrankungen

**Behandlung** s. Abschn. 12.2., 12.3., 12.4.1.

### 10.5. Husten

#### 10.5.1. Allgemeines

Husten ist bei einer akuten COVID‑19 Erkrankung ein häufiges Symptom und bleibt nach durchgemachter Erkrankung bei vielen Patient:innen länger als 3 Monate bestehen.

Bis 8 Wochen nach Beschwerdebeginn ist von einem postviralen Husten auszugehen, wo außer Anamnese und Status keine weitere Diagnostik notwendig ist. Voraussetzung ist das Fehlen von Warnzeichen.

Wenn aber der Husten über acht Wochen hinausgeht, handelt es sich um einen chronischen Husten – dieser muss weiter abgeklärt werden (s. dazu Leitlinie der DEGAM [143]). Dies gilt auch nach COVID‑19.

Entscheidend ist die Frage, ob es auch beschwerdefreie Episoden gibt, dann handelt es sich eher um rezidivierend auftretenden Husten, z. B. im Rahmen rasch aufeinander folgender Atemwegsinfektionen und dann wohl nicht um Husten aufgrund einer COVID-Infektion.

#### 10.5.2. Ausschluss potenziell gefährlicher Verlauf

Kombination mit Dyspnoe, Belastungsassoziation, Hämoptysen, Thoraxschmerz, Stauungszeichen.

#### 10.5.3. Zuordnung

##### Anamnese

- Beginn, Dauer und Art des Hustens, Vorgeschichte (Hinweise auf Asthma/COPD, andere vorbestehende Erkrankungen), weitere Symptome, Auswirkungen auf Leistungsfähigkeit und Nachtschlaf,

##### Klinische Untersuchung

- Auskultation von Herz und Lunge, Beurteilung des Oberbauchs (Reflux), s. dazu DEGAM Leitlinie Husten [143]

##### Weitere Untersuchungen

- Labor: BB, CRP bei Verdacht auf bakterielle Infektion
- Weitere Basisuntersuchungen: Lungenfunktion mit Broncholyse, Thoraxröntgen, ev. D‑Dimer, weitere Bildgebung (bei V. a. Pulmonalembolie – klinisch bzw. bei Vorliegen zusätzlicher Risikofaktoren)

#### 10.5.4. Procedere

##### Abwartendes Offenhalten

Wenn keine Hinweise auf eine organisch-strukturelle Erkrankung gefunden werden, und die Beeinträchtigung nur mäßig ist, kann zugewartet werden (Grenze: acht Wochen). Regelmäßige Kontrollen und die Wiedervorstellung bei Verschlechterung oder zusätzlicher Symptomatik werden vereinbart und dokumentiert.

##### Fachspezifische Abklärung

Bei belastender Symptomatik, die mit den angeführten Mitteln nicht erklärt und behandelt werden kann erfolgt die Zuweisung an die entsprechend den Ergebnissen aus Anamnese und Untersuchung geeignete Stelle.

#### 10.5.5. Weitere wichtige Differenzialdiagnosen

Alle internationale Leitlinien empfehlen nach einer Hustendauer von acht Wochen die Abklärung auf Neoplasien, rez. Lungenembolien, Fremdkörperaspiration und chronische Linksherzinsuffizienz mit Lungenstauung.

**Behandlung** s. Abschn. 12.4.3.

### 10.6. Thorakale Beschwerden

#### 10.6.1. Allgemeines

Thorakale Beschwerden treten bei Patient:innen noch Wochen nach akuter Infektion häufig auf. Bei thorakaler Post-COVID Symptomatik sind die Abklärung kardialer und pulmologischer Ursachen von Relevanz im Hinblick auf häufige Beteiligung dieser Organsysteme.

In der Pneumologie ist die Ätiologie des Thoraxschmerzes noch nicht gesichert, möglicherweise Folge der suszipierten autonomen Dysfunktion und Muskelschwäche im Rahmen von Long COVID bzw. des Post-COVID‑19 Syndroms. Beispielsweise gibt es bei physiotherapeutischen Untersuchungen Hinweise für eine Einschränkung der Zwerchfellmobilität sowie Hinweise auf eine Muskelschwäche der Atemmuskulatur.

In der Kardiologie steht bei der Abklärung von Thoraxschmerz die Detektion einer evtl. bestehenden Ischämie oder entzündlicher Herzerkrankung im Vordergrund.

#### 10.6.2. Ausschluss potenziell gefährlicher Verlauf

##### Hinweise für instabile Situation? (Z. B.: akutes Koronarsyndrom, Pulmonalembolie …)

- Zeichen des akuten Kreislaufversagens (Schockindex > 1)
- (unmittelbar vorangegangene) Synkope oder Kollaps
- Kaltschweißigkeit
- Aktuelle Ruhedyspnoe (Vorgehen s. Abschn. 9.4.)
- Ausgeprägte Angst der/des Betroffenen

##### Weitere „red flags“

- Fieber, starke Schmerzen, akut beeinträchtigte Atmung
- Typische Angina pectoris, insbesonders mit Crescendo Charakter

#### 10.6.3. Zuordnung

##### Anamnese

- Schmerzcharakteristik, Zeitpunkt des Auftretens, Dynamik, Atemabhängigkeit, Belastungsabhängigkeit, Bewegungsabhängigkeit, Leistungsminderung, Dyspnoe, Dyspepsie, Abhängigkeit von der Nahrungsaufnahme. Frage nach Verlauf der Akuterkrankung (Hinweise auf kardiale oder pulmonale Beteiligung?)

##### Klinische Untersuchung

- fokussierte Basisuntersuchung: Inspektion, Auskultation Herz und Lunge, Palpation des Thorax (Rippen, WS), dynamische Untersuchung des Oberkörpers und der oberen Extremitäten, Hautkolorit, Palpation des Oberbauchs

##### Weitere Untersuchungen

- RR, 12 Kanal-EKG (wenn kardiale Ursache klinisch nicht ausschließbar), Belastungstests, Thoraxröntgen (wenn pulmonale Ursache nicht ausschließbar). D‑Dimer, CRP, BB nur bei entsprechenden Hinweisen und Verdachtsmomenten.

#### 10.6.4. Procedere

##### Abwartendes Offenhalten

Nach sicherem Ausschluss von Red flags kann bei gutem klinischen Zustandsbild und fehlenden Hinweisen für reversible organspezifische Ursachen eine symptomatische Therapie entsprechend der vermuteten Pathogenese etabliert werden.

##### Fachspezifische Abklärung

Hinweise auf kardiale Ursachen:

- Abklärung nach etablierten Routinen (s. Abschn. 8.2.) Besonders zu beachten: Myokardischämie (EKG), entzündliche Herzerkrankungen

Hinweise auf pulmonale Ursachen:

- Abklärung nach etablierten Routinen (s. Abschn. 8.1.) – insbesondere bei gemeinsamem Auftreten mit Dyspnoe/Tachypnoe (s. Abschn. 10.4.):
- Akute Lungenembolie (D-Dimer), Pneumothorax, Pleuritis

Hinweise auf muskuloskelettale Ursachen:

- Bewegungsmangel (Isolierung, Kontaktreduktionsmaßnahmen)
- Patient:innen nach schwerem Verlauf (Hospitalisierung): Folge von Muskelabbau durch Immobilisierung

#### 10.6.5. Wichtige Differenzialdiagnosen ohne Zusammenhang mit COVID-19

Thoraxschmerzen können sehr häufig durch eine Reihe muskuloskelettaler Probleme bedingt sein, ebenso finden sich gastrointestinale (Reflux, Zwerchfellhernie, Ulcus, …) oder psychogene Ursachen. Kombinationen sind, wie immer, möglich.

**Behandlung** s. Abschn. 12.4.

### 10.7. Störungen der Hirnleistung

#### 10.7.1. Allgemeines

Es kann sich hierbei um Probleme in folgenden Bereichen handeln:

- Kognitive Symptome (Aufmerksamkeit, Exekutivfunktionen, deduktives Denken, Orientierung, Sprache, Gedächtnis, räumlich-visuelle und viso-motorische und -konstruktive Fähigkeiten)
- Gedächtnisprobleme („Arbeitsgedächtnis“ – Kurzzeitgedächtnis)
- Leichte kognitive Beeinträchtigungen im Sinne eines mild Cognitive Impairments (subjektive oder objektive Verschlechterung der prämorbiden Leistungsfähigkeit in einem oder mehreren Bereichen)
- Höhergradiges Ausmaß der kognitiven Beeinträchtigungen mit Krankheitswert

#### 10.7.2. Ausschluss potenziell gefährlicher Verlauf

- Plötzliche deutliche Verschlechterung oder akutes Neu-Auftreten,
- Auftreten multifokaler oder zentral neurologischer Symptome
- Hinweis auf reversible Ursache oder progrediente (Akut‑)Erkrankung?
- TIA bzw. Schlaganfall
- Akute Erkrankung (z. B. Enzephalitis)
- Hypoglykämie
- Akutes Delir
- Kognitive Verlangsamung im Rahmen einer schweren depressiven Episode? (Suizidale Gefährdung?)
- Denkhemmung im Rahmen einer schizophrenen Psychose

#### 10.7.3. Zuordnung

##### Anamnese (nach Ausschluss von „red flags“ – mit Fremdanamnese wenn möglich)

- Inkl. Medikamenten – und Drogenanamnese sowie psych. Exploration

##### Klinische Untersuchung

- Gezielter neurologischer Status

##### Basisdiagnostik

- Ausschluss systemischer Ursachen (z. B. Anämie, respiratorische Insuffizienz, Schilddrüsen-Unterfunktion)
- Objektivierung der Gedächtnisleistungsstörung: einfach durchführbare kognitive Tests
  - Minicog (3 Worte, Uhrentest),
  - MoCA (Montreal Cognitive Assessment)

#### 10.7.4. Procedere

##### Abwartendes Offenhalten

Dies ist nach Ausschluss potenziell gefährlicher Verläufe und reversibler Ursachen möglich, eine schrittweise Besserung (parallel mit der zunehmenden Normalisierung des zerebralen Glukosemetabolismus) ist in den meisten Fällen zu erwarten.

##### Fachspezifische Abklärung

- Bei Verdacht auf ein akutes zerebrales Geschehen oder akute reversible Ursache (vorhandene „red flags“) sollte eine entsprechende Abklärung entlang vorhandener Leitlinien inkl. zerebraler Bildgebung erfolgen.
- Die spezialisierte Abklärung wird bei gesicherter Neu-Manifestation/Therapieresistenz einer neurokognitiven Störung im Rahmen eines Long COVID-Syndroms empfohlen. Dann erfolgt eine weiterführende Differentialdiagnose nach den gültigen Leitlinien.
- Bei Persistenz der kognitiven Einschränkungen > 12 Wochen sollte eine Re-Evaluation (ggf. auch psychiatrisch) erfolgen

#### 10.7.5. Weitere wichtige Differenzialdiagnosen

##### In Zusammenhang mit COVID-19

- Depression
- Biorhythmus-Störung (Insomnie, fehlende Tagesstruktur)
- Andere organspezifische Ursachen als Folge von COVID‑19
- Folgen eines Delirs i.R. der schweren Erkrankung
- Irreversible Schädigungen z. B. i.R. einer Hypoxämie/ARDS

##### Ohne bekannte Assoziation mit COVID-19

- Somatische Ursachen z. B. Anämie, respiratorische Insuffizienz, Schilddrüsenfehlfunktion und metabolische Störungen,
- (Meningo‑) Enzephalitis anderer Ursache (u. a. ZNS-Infektionen z. B. Lyme-Borreliose)
- Unerwünschte Neben- und Wechselwirkungen von Medikamenten oder anderen Substanzen
- Klinische Manifestation einer subklinischen Gehirnerkrankung (mit und ohne Assoziation zu COVID‑19)
- Somatisierung

**Behandlung** s. Abschn. 12.4.

### 10.8. Schwindel („Dizziness“)

#### 10.8.1. Allgemeines

Primär ist zwischen einem gerichteten (Vertigo, Drehschwindel meist mit Richtungsangabe) oder ungerichteten Schwindel (Schwankschwindel) oder im Sinne leichter Benommenheit („dizziness“) zu unterscheiden. Im Zusammenhang mit COVID‑19 steht eher der ungerichtete Schwindel und die „Dizziness“, als die eigentliche Vertigo.

#### 10.8.2. Ausschluss potenziell gefährlicher Verlauf

- Relevante akute Beeinträchtigung – Vigilanz?
- Kurzanamnese bez. Schwindel – Charakter (Drehschwindel), Symptomdynamik (akut oder länger bestehend, plötzlich oder allmählich)
- Basiserhebung Vitalparameter (RR, HF rhythmisch?!, Sp02+AF, BZ)
- Auftreten gemeinsam mit anderen, multifokalen oder zentral neurologischen Symptomen?
  - Herzrhythmusstörung
  - TIA bzw. Schlaganfall
  - Neuronitis vestibularis
  - Benigner paroxysmaler Lagerungsschwindel
  - Hypoglykämie
  - Dehydratation

#### 10.8.3. Zuordnung

##### Anamnese

- Lageabhängigkeit, Bewegungsabhängigkeit, Provokationsmöglichkeit
- Differenzierung Gleichgewichtsstörung/Schwindel/Kreislauflabilität (s. Abschn. 10.9.)
- Sturz- bzw. (gerichtete) Fallneigung?
- Begleitende vegetative Symptomatik (Übelkeit, Erbrechen)
- Dauer: rezidivierend? persistierend? → bei Drehschwindel, mit begleitender Hörminderung oder zusätzlichen neurologischen Symptomen ist eine spezialisierte Abklärung notwendig.
- Erfassung der subjektiven und objektiven Beeinträchtigung

##### Klinische Untersuchung

- Auskultation, gezielter neurologischer Status
- Auflösung/Provokation durch Lagerungsmanöver möglich? (BPLS)

##### Basisdiagnostik

- EKG bei V. a. Herzrhythmusstörung, Blutdruckmessung inklusive liegend/stehend → „verkürzter Schellong“ (3 min aktiv stehend)

#### 10.8.4. Procedere

##### Abwartendes Offenhalten

- Wenn keine zusätzlichen Symptome und keine red flags vorliegen, die beschriebene Basisuntersuchung ohne Ergebnis bleibt und die Beschwerden den Alltag nicht wesentlich beeinflussen, kann beim ungerichteten Schwindel auf eine weiterführende Diagnostik verzichtet werden. Dies gilt insbesondere, wenn die Störung erst im Gefolge der COVID‑19 aufgetreten ist.

##### Fachspezifische Diagnostik („dizziness“)

- Bei objektivierbaren kognitiven Störungen, Störungen der Vigilanz oder des Gedächtnisses (Außenanamnese!) wird eine neurologische Begutachtung empfohlen, vor allem wenn diese nicht klar in Zusammenhang mit der abgelaufenen Infektion stehen
- Unspezifische „dizziness“ siehe auch Abschn. 10.9.
- Zum Abklärungsgang bei gerichtetem Schwindel (Vertigo) wird auf die entsprechenden Leitlinien verwiesen (s. DEGAM-Leitlinie „Schwindel, akut in der Hausarztpraxis“ [144]).

#### 10.8.5. Weitere wichtige Differenzialdiagnosen

In Zusammenhang mit COVID‑19

- Orthostatische Dysregulation, POTS (siehe 8.2 Kardiologie, 10.9 Kreislauflabilität)
- Somatisierendes Verhalten bei Long COVID: Angst‑/Panikattacken, Depressio mit Schwindel als Manifestation einer Somatisierung
- Unspezifische Gangunsicherheit bei Long COVID (s. 7.3. und 9.10.)

Ohne bekannte Assoziation mit COVID‑19

- Dehydratation, Anämie
- Verspannungen der Nackenmuskulatur
- Benigner paroxysmaler Lagerungsschwindel (BPLS)
- Kreislaufschwäche (siehe Abschn. 10.8.)
- Neuritis vestibularis, M. Menière
- Altersassoziierte unspezifische Schwindelsymptomatik
- Polypharmazie
- Angst/Panik
- Andere neurologische Ursachen (z. B. MS, Epilepsie, Polyneuropathie)
- Andere aktive/akute Infektionen oder Erkrankungen (Schilddrüsendysfunktion, Hypo- oder Hypertonie)

**Behandlung** s. Abschn. 12.4.5.

### 10.9. Kreislauflabilität

#### 10.9.1. Allgemeines

Kreislaufregulationsstörungen sind vor allem in den ersten Wochen nach der Infektion mit SARS-CoV‑2 nicht selten beschrieben [138]. Sie müssen von organischen, vor allem kardialen Krankheiten (auch: mögliche kardiologische Folgen von COVID‑19) abgegrenzt werden.

#### 10.9.2. Ausschluss potenziell gefährlicher Verlauf

- Plötzliche Einschränkung des Bewusstseins/Bewusstlosigkeit?
- Bei Synkope subjektive und objektive Beeinträchtigung (Vitalparameter: RR, HF, AF, spO2, Blutzucker und Körpertemperatur)
- Weitere „red flags“: Arrhythmie, Stauungszeichen, Hautkolorit blass oder zyanotisch, neurologische Symptomatik

#### 10.9.3. Zuordnung

##### Anamnese

- Differenzierung zwischen Schwindel, Gleichgewichtsstörung und Kreislauflabilität (orthostatische Komponente?)
- Prodrome? Unter körperlicher Anstrengung? Potenzielle vasovagale Auslöser bzw. situativ erklärbar?
- Dauermedikation? Neue Medikation? Trinkmenge? Alkohol und Drogen?

##### Klinische Untersuchung

- Auskultation Herz/Lunge, Blutdruckmessung (inkl. liegend/stehend! → „verkürzter Schellong“ – 3 min aktiv stehend), Zeichen für Dehydratation, Hinweise auf kardiorespiratorische Ursachen (Hautkolorit, Stauungszeichen, Anämie?)

##### Basisdiagnostik

- Basislabor inkl. BSG/CRP, EKG

#### 10.9.4. Procedere

##### Abwartendes Offenhalten

- Wenn Hinweise auf organische Ursachen in der Basisabklärung nicht gefunden werden, und die Beschwerden im Zusammenhang mit COVID‑19 aufgetreten sind, kann unter allgemeinen Maßnahmen (Bewegung, ausreichende Flüssigkeitszufuhr etc.) die Besserung abgewartet werden. Die Patient:innen sollten informiert werden, sich bei Verschlechterung oder zusätzlich auftretenden Symptomen wieder vorzustellen.

##### Fachspezifische Abklärung

- Sind die Kreislaufregulationsstörungen Alltags- bzw. arbeitsrelevant, kann eine Abklärung nach 3–4 Wochen erwogen werden, bei Persistenz über 12 Wochen hinaus ist die Re-Evaluierung jedenfalls empfohlen.
- Bei Hinweisen auf eine strukturelle bzw. Organsystem bezogene Erkrankung (neurologisch, pulmologisch, endokrinologisch, kardiologisch…) ist eine fachspezifische Abklärung entlang der üblichen Leitlinien empfohlen (*EbM-Guidelines Synkopen: Ursachen und Abklärung, S1-Leitlinie DGN – Synkopen*).

#### 10.9.5. Weitere wichtige Differenzialdiagnosen

In Zusammenhang mit COVID‑19 z. B.:

- Postinfektiös: Postural Tachykardia Syndrom (POTS): Orthostaseintoleranz, Tachykardie bei Orthostase, Palpitationen, Schwindelgefühl, Sehstörungen, Präsynkopen und Belastungsintoleranz sind typisch
- Kardiale Genese nach COVID‑19 (aber auch unabhängig davon möglich):
  - Herzrhythmusstörungen
  - Myokarditis
  - Herzinsuffizienz
- Postinfektiöse Thyreoiditis
- Postinfektiöse Fatigue

Ohne bekannte Assoziation mit COVID‑19 z. B.:

- Dehydratation bzw. Elektrolytstörungen (z. B. Hyponatriämie), Anämie
- Andere aktive Infektionen
- Unerwünschte Neben- oder Wechselwirkung bei Polypharmazie
- Somatisierendes Verhalten im Rahmen einer psychischen Erkrankung (z. B. Angst/Depressio)
- Schilddrüsendysfunktion
- Multifaktoriell, unspezifisch (siehe auch: *EbM Guidelines: Stürze beim älteren Menschen*)

**Behandlung** s. Abschn. 12.2., 12.4.5.

### 10.10. Schlafstörung

#### 10.10.1. Allgemeines

Post-COVID‑19 werden Schlafprobleme häufig beschrieben. Die Ursachen sind vielfältig und bisher nicht sicher spezifisch mit der Virus-Erkrankung assoziiert [8].

#### 10.10.2. Ausschluss potenziell gefährlicher Verlauf

**Red flags: **Ausgeprägt depressive, manische oder psychotische Komponente, schädlicher Medikamenten- oder Drogenkonsum

#### 10.10.3. Zuordnung

##### Anamnese

- subjektiv zeitlicher Zusammenhang mit COVID‑19 oder Verstärkung vorbestehender Beschwerden.
- Ein- oder Durchschlafstörung, Störung des Schlafrhythmus, Tagesmüdigkeit
- Atemaussetzer – auch ohne Schnarchen (Fremdanamnese), Schlaf tagsüber
- Fragen nach negativen Gedanken, Gedankenkreisen, Angst, Aufgeregtheit. Gefragt wird auch nach Schlafgewohnheiten, Lebensstil incl. Medikamenten, psychosozialen belastenden Faktoren mit oder ohne Zusammenhang mit der Pandemie, und störenden körperlichen Sensationen (Juckreiz, Schmerzen, restless legs, Atemnot etc.). Sorgen bezüglich eines eventuell behindernden Verlaufs von COVID‑19, s. dazu Leitlinie Schlafstörung der DEGAM, Anwenderversion [145].

##### Klinische Untersuchung

- HNO-Bereich, BMI, RR

##### Weitere Untersuchungen

- nur bei Hinweisen auf somatische Ursache: Schlaftagebuch, Schlaflabor

#### 10.10.4. Weitere wichtige Differenzialdiagnosen

- Vorbestehende schlafmedizinische Probleme (Einschlafstörung, Durchschlafstörung, nicht erholsamer Schlaf, Tagesmüdigkeit) oder psychische Probleme können, im Rahmen der Pandemie, verstärkt werden oder stärker empfunden werden.
- Selten: Somatische Ursachen (Hinweise aus der Anamnese). Siehe dazu S3-Leitline der Deutsche Gesellschaft für Schlafforschung und Schlafmedizin e. V. (DGSM) [146].

**Behandlung **s. Abschn. 12.2., 12.3.

### 10.11. Nerven- und Muskelaffektionen

#### 10.11.1. Allgemeines

Grundsätzlich stellt die Trias Myalgien, Fatigue und Hyper-CK-ämie in COVID‑19-Kohorten die häufigste Form (40–70 %) einer Skelettmuskelaffektion dar [66].

Myalgien kommen im Akutstadium der COVID-Infektion häufig (~ 20 %) vor. Ca. 50 % der Patient:innen erholen sich von ihren Myalgien innerhalb von wenigen Tagen. Längerfristig können in ca. 6 % muskuloskelettalen Beschwerden, sowie Fatigue und geringe Bewegungsbelastbarkeit persistieren [66].

#### 10.11.2. Ausschluss potenziell gefährlicher Verlauf

- Sensible Defizite, motorische Defizite, Paresen, Bewusstseinseintrübungen, kognitive Defizite, Wesensveränderung, Agitation & Delir, Sehverschlechterung, Bewegungsstörungen, Sprachstörungen, Schluckstörungen, Koordinationsstörungen, (nicht-)konvulsive Anfälle, Urin- und Stuhlinkontinenz, kardiovaskuläre Komplikationen, Arrhythmien, respiratorische Insuffizienz
- Blasse, kalte Extremität, bei deutlich unterschiedlichen Fußpulsen, Schmerzen oder Schwellung, rote, dicke Extremität im Seitenvergleich

#### 10.11.3. Zuordnung

##### Anamnese

- Zeitpunkt des Auftretens, Lokalisation, Dauer, Dynamik, Belastungsabhängigkeit, Provokations‑/Linderungsfaktoren, Ansprechbarkeit auf Schmerztherapie
- Bewegungsanamnese
- Weitere Symptome (besonders Fatigue, chronische Schmerzen)
- Vorerkrankungen, Spitalsaufenthalt, Familienanamnese, Medikamente (z. B. Statine), Alkohol, andere toxische Einwirkungen

##### Klinische Untersuchung

- zur Objektivierung der subjektiv empfundenen Beschwerden:
- Je nach Anamnese: Suche nach Zeichen zugrundeliegender Erkrankungen – (Differenzialdiagnosen s. weiter unten)
- Sorgfältige klinische Untersuchung inkl. Motorik, Sensibilität, Kraft, Reflexe und Durchblutung im Seitenvergleich

##### Weitere Untersuchungen

Labor: BB, BZ, ev. CRP-, D‑Dimer-Schnelltest bei konkreten klinischen Hinweisen, ev. CK

- Weitere Laborwerte sowie Anwendung von bildgebenden und apparativen Verfahren je nach Verdachtsdiagnose

#### 10.11.4. Procedere

##### Abwartendes Offenhalten

- Wenn eine erste Abklärung keine Hinweise auf neurologische/internistische/orthopädische Pathologien erbringt, kann in Absprache mit den Patient:innen mit einer weiterführenden Abklärung zugewartet werden (wichtig ist das Fehlen von „red flags“ und auch sonst unauffälligen Untersuchungsergebnissen).
- Bei Assoziation mit COVID‑19 sollten die Betroffenen darüber aufgeklärt werden, dass ihre Beschwerden im Zusammenhang mit COVID‑19 häufig vorkommen können, aber in den meisten Fällen wieder remittieren.
- Eine klinische Kontrolle bei der Hausärztin/beim Hausarzt wird je nach Zustandsbild empfohlen. Bei Persistenz über 12 Wochen sollte zur Re-Evaluierung führen. Auf die Notwendigkeit der Wiedervorstellung bei Verschlechterung ist dokumentiert hinzuweisen!

##### Fachspezifische Abklärung

- Critical-Illness-Neuro-Myopathie (ICU – Aufenthalt? Dauer ICU-Aufenthalt? Muskelatrophien? Areflexie?)
- Rhabdomyolyse (wenn CK > 10.000 U/L, Nierenverschlechterung, Harn braun)
- Guillain-Barré-Syndrom (Von distal symmetrisch aufsteigende Par- und Hypästhesien bis hin zu schweren Tetraparesen. Auch bilaterale Fazialisparesen, Augenmuskelparesen oder Miller-Fisher-Syndrom. Respiratorische Insuffizienz)
- Konus‑, Cauda-Syndrom, Reithosenanästhesie
- TVT, PAVK

Zur weiteren spezialisierten Abklärung einer unklaren Myalgie siehe „Diagnostik und Differenzialdiagnose bei Myalgien“, S1-Leitlinie der Deutschen Gesellschaft für Neurologie (DGN) [73].

#### 10.11.5. Weitere wichtige Differenzialdiagnosen ohne Zusammenhang mit COVID-19

- Persistenz der Schwäche und Muskelatrophie nach Spitalsaufenthalt oder ICU
- (Verschlechterung eines vorbestehenden) muskuloskelettalen Schmerz-Syndroms, Wirbelsäulen‑, Gelenksbeschwerden
- Arthritis, Arthrose, rheumatologische Erkrankungen
- Polyneuropathie, z. B. im Rahmen von Diabetes mellitus Typ II, Hypothyreose, Alkoholabusus, Vit. B12-Mangel (selten B6, B1, E), hereditär, bei HIV-Infektion
- Myositis, Dermatomyositis, systemische Autoimmunerkrankung
- Medikamentennebenwirkungen (insbesondere Statine, Ciprofloxacin, Bisphosphonate, Aromatasehemmer, Fibrate)
- Myopathien, Schilddrüsenerkrankung, Nebenniereninsuffizienz, Vitaminmangel, Leber- oder Nierenerkrankungen, Elektrolyt-Verschiebungen, Krebserkrankungen
- Nerven- und Muskelaffektionen im Rahmen des chronischen Erschöpfungssyndroms (ME/CFS), Fibromyalgie-Syndrom, Restless-Leg-Syndrom
- Nerven- und Muskelaffektionen im Rahmen von Long-COVID
- Verschlechterung der körperlichen Belastbarkeit wegen z. B. Bewegungsmangel/vermehrtes Sitzen während der Pandemiemaßnahmen und psychischen Faktoren

**Behandlung** s. 12.2, 12.4.6

## 11. Follow-up und Monitoring

In internationalen Leitlinien werden unterschiedliche Konzepte vorgeschlagen. Eine Synopse der für die Primärversorgung relevanten Leitlinienempfehlungen findet sich in Tab. 4 (Screening und Monitoring).

**Table 4:** 

	Long COVID/PASC (Post-acute sequelae of SARS-CoV‑2 infection) MEDIX, CH	Long COVID‑19: Proposed Primary Care Clinical Guidelines for Diagnosis and Disease Management Autorinnen und Autoren der CAMFiC long COVID‑19 Study Group, ES	National guidance for post-COVID syndrome assessment clinics NHS, UK	Managing the long-term effects of COVID‑19 SIGN, UK	Caring for adult patients with post-COVID‑19 conditions RACGP, AU	SARS-CoV‑2, COVID‑19 und (Früh‑)Rehabilit ation AWMF, DE
Screening	0	3 Termine in PV: **4 Wochen** nach pos.Test/Symptombeginn **8 Wochen** **12 Wochen**	Amb: keines Hospitalisiert: 12 Wochen n. E. N. ICU: Pfad intramural –12 Wochen n. E.	Information (bei Diagnosestellung), kein Screening	Hosp: telemedizinisch kurz n. E., Folgetermin 6–8 Wochen (Präsenz)	0
Monitoring	Anlassbez. Keine red flags: Beratung Red flags: erweiterte Abklärung	Lt. Behandlungspfaden	Amb:bei Beschwerden > 4 W. PV Weiterleitung nach Eval. Hosp:12 n. E.	Individualisiert, anlassbez.	Ältere, Menschen m. Beeintr. proaktiv Andere: Anlassbezogen	Keine Angaben
Management	Empf. f. Selbstmanagement, Unterstützung	Diagnose Post-Covid 19: Vorstellung im spezialisierten Bereich	Post-Covid Kliniken: über Allgemeinmedizin o.a. Ärzt:innen, ab 4 Wochen nach Erkrankungsbeginn	Information bei Diagnose Akuterkrankung	–	Fachärztlich orientiert

Die folgenden Empfehlungen orientieren sich an diesen Leitlinienempfehlungen, wurden aber an die Gegebenheiten des österreichischen Gesundheitssystems und die rezenten Ergebnisse aus Studien zum COVID‑19 Verlauf angepasst [11].

*Anmerkung: auch diese Empfehlungen beziehen sich* **nicht** *auf Personen nach Intensivüberwachung.*

### 11.1. Untersuchung nach Ende der Quarantäne

Die Richtlinien zur Entlassung aus der Isolierung orientieren sich bisher vor allem an der Kontagiosität [147]. Österreichischen Zahlen [148] zufolge ist bei Ende der Quarantäne erst weniger als ein Drittel der Betroffenen beschwerdefrei, und mehr als die Hälfte leidet noch an zwei oder mehr Symptomen. Ähnliche oder noch höhere Zahlen finden sich in der internationalen Literatur [11]. Anders als bei Wiederaufnahme der Berufstätigkeit nach anderen Erkrankungen, ist nach COVID‑19 bisher keine Gesundschreibung vorgesehen, da es sich nicht um einen ärztlich verordneten Krankenstand handelt, sondern um eine behördliche Absonderung.

#### Empfehlung

Eine primärärztliche Untersuchung vor Wiederaufnahme von Alltags- und Berufstätigkeit erscheint angemessen – vor allem, wenn es sich um körperlich anstrengende Tätigkeiten handelt. Im Rahmen einer solchen Begutachtung können einerseits organische Ursachen ausgeschlossen werden, andererseits ist es möglich, den Wiedereinstieg sowie ev. ein Monitoring situationsgerecht zu planen.

Für hospitalisierte Patient:innen sollte im Zuge der Entlassung ein Follow-up je nach individueller Situation empfohlen werden.

### 11.2. Anhaltende Symptome von COVID-19 > 4 Wochen

Ohne Hospitalisierung: Wenn nach Ende der Akuterkrankung weiterhin Symptome bestehen, empfehlen die meisten Leitlinien eine Begutachtung im Bereich der Primärversorgung [2, 8, 149]. Dabei sollen spezifische Ursachen entweder ausgeschlossen oder abgeklärt und behandelt werden. Bei funktionellen Beschwerden, erfolgt eine Einstufung der Beeinträchtigung und es wird mit den Betroffenen gemeinsam ein Plan erarbeitet (s. dazu Kap. 13). Die meisten Leitlinien warnen vor Überdiagnostik. Eine routinemäßige Vorstellung nach 8 Wochen erscheint angesichts der interindividuell sehr unterschiedlichen Verläufe und der hohen Systembelastung nicht sinnvoll. Wir sehen ein individuell angepasstes Monitoring als angemessen an.

Mit Hospitalisierung: Folgeuntersuchungen wie bei Entlassung vereinbart, bzw. bei Verschlechterung oder Auftreten neuer Symptome im allgemeinärztlichen Bereich bzw. bei bekannten organsystembezogenen Krankheitsfolgen bei der Spezialistin/dem Spezialisten. In jedem Fall und unabhängig von Symptomen wird spätestens 12 Wochen nach Entlassung eine Follow-up Untersuchung empfohlen [8].

### 11.3. Post COVID-19: Symptomatik > 12 Wochen

Ohne Hospitalisierung: Bei anhaltenden oder wiederkehrenden Beschwerden über 12 Wochen nach Erkrankungsbeginn hinaus, bei erheblichen Beschwerdeausmaß auch schon früher, sollte eine Re-Evaluierung und gegebenenfalls die weitere Abklärung im spezialisierten Bereich erfolgen. Ab einer Einstufung als Grad 2 nach der Klok Skala (Abb. 4) sollte die Einleitung einer ambulanten oder auch stationären Rehabilitationsbehandlung überlegt werden, vor allem dann, wenn keine klare Besserungstendenz ersichtlich ist.

Nach Hospitalisierung: Eine regelhafte Untersuchung nach 12 Wochen ist in vielen Leitlinien empfohlen (s. oben).

#### Empfehlung

Patient:innen sollte geraten werden, bei Beschwerdepersistenz über 4 Wochen jedenfalls eine Abklärung innerhalb der hausärztlichen Primärversorgung in Anspruch zu nehmen. Bei Symptomen, die länger als 12 Wochen anhalten, sollte eine Re-Evaluierung und gegebenenfalls die gezielte weiterführende Abklärung angeboten werden.

## 12.Behandlung

### 12.1. Allgemeine Maßnahmen

Belastbare Evidenz aus Studien zur Behandlung von Long COVID Symptomen ist noch nicht verfügbar, Interventionelle Studien sind uns bisher nicht bekannt [2].

#### Drei zentrale Ansätze werden in den meisten Leitlinien angeführt

- Wenn eine dem Symptom zugrunde liegenden Pathologie identifiziert werden kann, erfolgt die Behandlung entsprechend diesem Befund nach den üblichen Regeln und Leitlinien.
- Ansonsten erfolgt die symptomatische Behandlung, sowie Begleitung und Unterstützung bis zur für die meisten Patient:innen sehr wahrscheinlichen völligen Wiederherstellung.
  - s. dazu Abschn. 11.4.
- Der dritte therapeutische Ansatz ist die Unterstützung der Wiedereingliederung bzw. die Rehabilitation (s. Kap. 13)

#### Therapeutische Impfung

Die Datenlage zu einer therapeutischen Vakzinierung ist derzeit noch unzureichend. Diese soll daher Studien vorbehalten bleiben.

#### Grundsätze

- Jeder Behandlungsentscheidung geht die sorgfältige Abklärung der Ätiologie voraus (Kap. 8)
- **Behandlung spezifischer Erkrankungen** (Aggravierung vorbestehender Komorbiditäten oder neu aufgetretene organische Störungen) je nach Situation im hausärztlichen oder spezialisierten Setting, oder in Kooperation
- **Behandlung funktioneller Störungen**, Betreuung und Monitoring vorzugsweise im Team der hausärztlichen Primärversorgung [8, 9]. Über die grundsätzlich gute Prognose funktioneller Störungen sollten die Patient:innen informiert werden. Eine Objektivierung des individuellen Leidensdrucks und des Ausmaßes der Beeinträchtigung bildet eine weitere Entscheidungsgrundlage für die Wahl der Behandlungsstelle. Die Post-COVID‑19-Skala des funktionellen Status (Abb. 4) als validiertes Tool hilft bei der Bewertung der bestehenden Leistungseinschränkung.
  - Auch wenn kein Substrat gefunden werden kann, und keine wesentliche funktionelle Einschränkung vorliegt, soll eine Copingstrategie gefunden werden, ebenso wie anlassbezogene oder auch terminlich fixierte Kontrollen.
  - Die Ermittlung und Berücksichtigung psychosozialer Umstände, ob durch die Infektion oder die Pandemie und ihre Folgen bedingt, oder auch davon unabhängig bestehend, ist essentiell und Teil guter hausärztlicher Praxis.
  - Zuziehung von Gesundheitsberufen (Physiotherapie, Psychotherapie, Ergotherapie…) situationsabhängig.
  - Ambulante oder stationäre Rehabilitationsmaßnahmen sollten ab einer Beeinträchtigung 2. Grades auf der Post-COVID‑19-Skala des funktionellen Status (s. Abschn. 8.3.1., Abb. 4) überlegt werden. Wenn die Beschwerden mehr als 3 Monate andauern und keine klare Besserungstendenz ersichtlich ist, bei starker Beeinträchtigung auch schon früher.
    Je schwerer der Verlauf der Akuterkrankung, desto wahrscheinlich wird die Notwendigkeit einer strukturierten Rehabilitation.
  - Wenn sich eine langfristige Problematik zeigt, ev. Anbindung an Selbsthilfegruppen.

#### Empfehlung

Behandlung, Begleitung und Monitoring sollten jedenfalls erfolgen, auch wenn die Symptomatik unklar erscheint, und/oder ein kausaler Zusammenhang mit COVID‑19 nicht gesichert werden kann.

Das Behandlungskonzept wird individuell geplant: entsprechend den Ergebnissen der Abklärung, und in Zusammenschau mit subjektivem Leidensdruck und den Vorstellungen und Möglichkeiten der Betroffenen [9].

### 12.2. „Pacing“

Pacing ist ein personenzentriertes Verfahren, das Patient:innen ermöglichen kann, ihre körperliche, kognitive und emotionale Energie innerhalb individueller Grenzen zu steuern, durch sorgfältige Planung, wo und wie die verfügbare Energie eingesetzt werden kann. Es ist ein Instrument, um eine post-exertionale Erschöpfung zu verhindern und/oder zu reduzieren. Aktivitätsprotokolle sowie Herzfrequenz- und Aktivitätsmonitore können verwendet werden, um den Patient:innen zu verdeutlichen, wann sie ihre spezifischen Energiegrenzen überschreiten. Trotz solcher Hilfsmittel ist das Pacing eine anspruchsvolle Aufgabe und Rückschläge sind unvermeidlich, zumal die Toleranzgrenze für Aktivität interindividuell und auch intraindividuell von Tag zu Tag variieren kann.

Dies gilt auch für alle Bereiche: körperliche und kognitive Leistungsfähigkeit, emotionale und mentale Belastbarkeit [150–152].

- Langsame Wiederaufnahme von Alltagstätigkeiten und -belastungen auf niedrigstmöglichem Niveau
- Steigerung des Niveaus, wenn die jeweilige Belastung gut toleriert wird (subjektiv und gemessen durch RR, HF, SpO2).
- Bei Verschlechterung der Symptome: Pause und Rückkehr zum absolvierbaren Niveau nach Abklingen der akuten Beschwerdesymptomatik („Symptom-titriertes Training“)
- Evaluation einer Rehabilitationsmöglichkeit bzw. – Notwendigkeit
  - Ambulante Rehabilitation? Physiotherapie?
  - Ergotherapie, Klinische Psychologie

#### Empfehlung

Personen, die infolge einer Infektion mit SARS-CoV‑2 an Müdigkeit und/oder Leistungsminderung in physischer, mentaler oder emotionaler Hinsicht leiden, sollen über die Methode des Pacings eingeführt und entsprechend monitiert werden.

### 12.3. Coping

Wesentlich ist die Vermeidung von unnötiger Angst und Unsicherheit auf Seiten der Betroffenen.

Ein individueller ganzheitlich orientierter Behandlungsplan sollte immer dann gemacht werden, wenn die Symptome als belastend empfunden werden. Er kann folgende Bereiche umfassen (nach NICE [8]):

- Selbstmanagement der Symptome („was hilft mir“)
- Selbstkontrollen (Tagebuch, Pulsoxymeter etc.)
- Anlaufstellen
- Unterstützungsmöglichkeiten („wer hilft mir“ – familiär, weitere Umgebung, professionell)
- Salutogenese („welche sind meine gesunden Anteile, was kann ich gut, wie und wo fühle ich mich wohl“)
- Empfehlung von verlässlichen und Warnung vor unverlässlichen Internetquellen.

#### Empfehlung

Angemessene Information darüber, dass in den meisten Fällen eine Besserung der funktionellen Beschwerden von selbst eintreten wird, ist essenziell, ebenso aber ein Ernstnehmen des individuell empfundenen Leidensdrucks.

Die Vermeidung einer Fixierung auf die Symptome sowie von Übermedikalisierung (Von Überdiagnostik bis Übertherapie) steht im Vordergrund [9].

### 12.4. Symptomorientierte Behandlungsoptionen

Im Folgenden werden die nach COVID‑19 am häufigsten beschriebenen Symptome angeführt, und Empfehlungen, meist aus der Erfahrungsmedizin stammend, zusammengefasst.

#### 12.4.1. Dyspnoe (zur Differenzialdiagnostik s. Abschn. 10.4.)

Der Einsatz von oralem Kortison muss im Einzelfall und nach Indikationsstellung durch Pneumologen bei stagnierender Besserung und einer Bildgebung passend zu einer organisierenden Pneumonie erwogen werden [153].

Für den Einsatz einer antifibrotischen Therapie gibt es aktuell ebenso keine Evidenz.

Inhalierbare Kortikosteroide oder Betamimetika werden dann empfohlen, wenn es Hinweise auf eine obstruktive Komponente und/oder eine bronchiale Hyperreagibilität gibt (Anamnese, Klinik Spirometrie) und die Kriterien lt. Leitlinien dafür erfüllt sind.

Eine milde bis moderate Dyspnoe ist nach COVID‑19 nicht selten, und remittiert normalerweise auch ohne Behandlung nach einigen Wochen. Ein Versuch mit dem beschriebenen Pacing lohnt sich.

Das Erlernen von *Atemtechnik* (Infobox 1) kann Erleichterung schaffen.

##### Infobox Atemtechnik

Ca. 80 % der Atemarbeit wird vom Zwerchfell geleistet. Im Gefolge einer Erkrankung oder allgemeiner Abbauvorgänge kann das Atemmuster verändert sein. Die Zwerchfellmobilität kann reduziert sein, und der Einsatz der Atemhilfsmuskulatur daher verstärkt. Daraus resultieren: flachere Atmung, raschere Ermüdung, Kurzatmigkeit, und erhöhter Energieverbrauch. Die Methode der Atmungskontrolle soll das Atemmuster normalisieren, den Einsatz der Atemmuskulatur (inkl. Zwerchfell) effizienter machen, und den Energieaufwand damit reduzieren. Dies reduziert auch die Irritation der Atemwege, die Ermüdung, und die Kurzatmigkeit.

Die Patient:in soll eine Sitzhaltung einnehmen, in der sie sich abstützen kann, und langsam atmen: es soll möglichst durch die Nase ein- und durch den Mund ausgeatmet werden. Dabei sollen Brust und Schultern bewusst entspannt bleiben, und die Bewegung des Bauches ungehindert möglich sein. Es sollte ein Verhältnis von 1:2 zwischen Ein- und Ausatmungsdauer angestrebt werden. Diese Übung kann über den Tag verteilt mehrmals wiederholt werden, Übungsdauer jeweils 5–10 min, oder auch länger. Andere Atemtechniken, wie z. B. die Zwerchfellatmung, langsames und tiefes Atmen, Atmen mit gespitzten Lippen, Yoga- oder Buteyotechniken können nach Indikationsstellung durch Spezialisten und unter Anleitung speziell ausgebildeter Personen eingesetzt werden ([9]; eigene Übersetzung).

Anmerkung d. Autoren: Die beschriebene Technik kann als „4711-Methode“ einprägsam vermittelt werden: 4 s ein-, 7 s ausatmen, 11 Wiederholungen.

Symptomatik und Leistungsfähigkeit von Patient:innen nach schwerem Verlauf bessern sich im Rahmen einer post-akuten frühen, multimodalen Rehabilitation [154]. Weiters ist eine multimodale Rehabilitation auch ohne Nachweis einer Organpathologie bei Long COVID empfohlen, auch wenn Evidenzlage für die Interventionen noch unzureichend ist [138].

#### 12.4.2. Leistungseinschränkung/Fatigue (zur Differenzialdiagnostik s. Abschn. 10.1., 10.2.)

- Wenn keine organische Erkrankung als Ursache für die Leistungseinschränkung gefunden wird, ist auch hier die Prognose in den meisten Fällen gut. Wie bei etlichen anderen Infektionskrankheiten bessert sich die Symptomatik im Laufe einiger Wochen, bei Persistenz > 12 Wochen ist eine Reevaluation notwendig.
- Wenn auch eine Re-Evaluierung keine zugrundeliegende Ätiologie erbringt (Abschn. 9.2.) und die Beeinträchtigung als Klok-Skala Grad 2 nach der Post-COVID‑19-Skala des funktionellen Status (Abb. 3) oder mehr eingestuft wird, sollten rehabilitative Maßnahmen erwogen werden (s. Abschn. 12.3.) und auf die Evaluierung der psychischen und sozialen Situation nicht vergessen werden. Siehe dazu auch Fatigue Assessment Scale (Tab. 2).
- Unbedingt erforderlich ist die integrierte psychosoziale und somatische Betreuung, um Therapien ohne rationalen Hintergrund zu vermeiden. Nur nachgewiesene Mängel sind zu substituieren (z. B. Vit D Mangel), eine Übermedikalisierung ist zu vermeiden, da Evidenzen fehlen und meist Placebo-Effekte vorherrschend sind. Ausreichend Erholung ermöglichen, Überforderungen und Überlastungen sind Eckpfeiler. Empfohlen wird eine Methode, die unter „Pacing“ bekannt wurde [151], Details s. Abschn. 12.2.
- Es gibt gute Erfahrungen mit Trainingstherapie bei postinfektiöser Fatigue [2, 151]. Immer wieder werden aber auch Verschlechterungen nach körperlicher Anstrengung beobachtet. Persönliche Leistungsgrenzen müssen daher grundsätzlich respektiert werden. Ein NICE-Statement dazu wird für August 21 erwartet. Zum Selbstmonitoring und -management (Pulsmesser, Pulsoxymeter, Symptomtagebücher) sind geeignete Methoden, um individuelle Belastungsgrenze zu erkennen. Als hilfreich beschrieben sind auch kognitiv-behaviorale Verfahren [151]. Details s. Abschn. 12.2.

#### 12.4.3. Husten (zur Differenzialdiagnostik s. Abschn. 10.5.)

Empirisch und analog zu den Empfehlungen bei postinfektiösem Husten kann ein Therapieversuch mit einem inhalativen Steroid eingeleitet werden. Beta-2-Sympathomimetika werden nur bei Indikation und entsprechend der Leitlinien eingesetzt. Bei fehlender Besserung nach einer Kurzintervention weiterführende Abklärung und spezifische Therapie durch den Lungenfacharzt (s. Abschn. 8.1.). Darüber hinaus ist bei anhaltendem Reizhusten ohne Substrat das Erlernen von Atemtechnik eine Option (Infobox 1). Auch eine logopädische Therapie wäre anzudenken.

#### 12.4.4. Sensori-neurale Riechstörungen (zur Differenzialdiagnostik s. Abschn. 10.3.)

Die Therapie COVID‑19 bedingter Riechstörungen unterscheidet sich nicht von der Therapie sensori-neuraler Riechstörungen anderer Genese. Da die Störung nach COVID‑19 aber recht häufig ist, wird sie an dieser Stelle ausführlicher beschrieben.

Aufgrund der kontinuierlichen Erneuerung der Riechnervenzellen besteht eine hohe Regenerationsfähigkeit des Riechvermögens. Als eine in Studien wirksam bewertete Therapieform hat sich die *Durchführung eines strukturierten Riechtrainings* erwiesen [155]. Dazu sollen die betroffenen Patient:innen zumindest zweimal täglich über einen Zeitraum von jeweils 2 min an insgesamt 4 verschiedenen Duftölen riechen. Die entsprechenden Fläschchen können in der Apotheke oder Drogerie von den Patient:innen selbst ohne Rezept gekauft werden (es eignen sich unterschiedliche Duftqualitäten, wie blumig, fruchtig, würzig, harzig, rauchig, etc.). Aufgrund der komplexen zentralen Prozesse bei der Wahrnehmung von Duftstoffen, scheint die bewusste und konzentrierte Durchführung des Trainings wichtig zu sein. Es soll dadurch zu einer beschleunigten Re-Organisation der Verbindungen der Riechnerven im Riechkolben und in weiterer Folge in höheren zentralen Hirnarealen in Stirn- und Schläfenlappen kommen, die für die Wahrnehmung von Duftstoffen verantwortlich sind. Das Riechtraining sollte zumindest für 6–9 Monate durchgeführt werden [156, 157].

Immer sind die betroffenen Patient:innen darauf hinzuweisen, auf eine Nikotin-Karenz zu achten, da Rauchen die olfaktorische Sensitivität vermindert [158].

Es existieren derzeit keine evidenz-basierten medikamentösen Therapien bei sensori-neuralen Riechstörungen[97].

Auf jeden Fall sollten die Patient:innen auf mögliche Gefahren der Riechstörung, wie die verspätete Wahrnehmung von verdorbenen Lebensmitteln, Feuer, Verbranntem, oder austretendem Gas hingewiesen werden [159]. Auch ist auf entsprechende Körperhygiene und bei Bedarf auch auf die Installation von Rauchmeldern zu achten.

Patientenblatt s. Abb. 2: *Riechtraining*

#### 12.4.5. Orthostatische Dysregulation (zur Differenzialdiagnostik s. Abschn. 10.8., 10.9.)

- Kompressionstherapie (bis hin zum Bauchnabel)
- Salzreiche Kost (Steigerung auf 8–10 g/d) und Flüssigkeit (3 l/d) bei fehlenden Kontraindikationen
- Aktivierende Bewegung und Grundlagenausdauertraining nach individueller Verträglichkeit

#### 12.4.6. Nerven- und Muskelschmerzen (zur Differenzialdiagnostik s. Abschn. 10.11.)

Die medikamentöse Therapie bei unspezifischen Nerven- und Muskelschmerzen ist zunächst rein symptomatisch, mittels (kurzzeitig) NSAR, Paracetamol und Metamizol. Diese soll die schrittweise Wiederaufnahme der täglichen körperlichen Aktivität unterstützen. Bei Therapieresistenz/Persistenz ist die Re-Evaluierung und ev. Kooperation mit Kolleg:innen der Sonderfächer zu empfehlen.

Patient:innen mit anhaltenden Beschwerden profitieren besonders von physikalischen Therapien/Physiotherapie.

Pacing-Ansatz beachten, um post-exertionale Malaise zu verhindern, welche zur Chronifizierung der Erkrankung führen kann, s. Beh. Allgemeines Pacing.

#### 12.4.7. Hauterkrankungen (zur Differenzialdiagnostik s. Abschn. 8.5.)

- Bei urtikariellen Exanthemen niedrig-dosierte systemische Kortikosteroide und Antihistaminika
- Bei konfluierenden, erythromatösen/makulopapulösen/mobiliformen Exanthemen topische und systemische Kortikosteroide
- Bei papulovesikulösen Exanthemen wait-and-see
- Bei akralen Pernionen wait-and-see
- Bei Livedo reticularis/racemosa Hautveränderungen wait-and-see
- Bei vaskulitischen Hautveränderungen topische und systemische Corticosteroide
- Bei durch das Coronavirus getriggerten anderen dermatologischen Erkrankungen Einleitung einer Therapie entsprechend den Leitlinien der einzelnen Dermatosen
- Bei Effluvium symptomatische Therapie wie z. B. topisches Minoxidil

#### 12.4.8. Weitere funktionelle Störungen und Symptome (zur Differenzialdiagnostik s. Kap. 9 und 10)

Es gelten die Grundsätze, die auch sonst für die symptomatische Behandlung dieser Symptome gelten.

#### 12.4.9. Psychiatrische Krankheitsbilder (zur Differenzialdiagnostik s. Abschn. 8.6.)

Es gibt keine spezifischen Therapieempfehlungen für die Behandlung psychischer Störungen im Kontext von Long COVID. Es gelten die üblichen Therapieempfehlungen, gegebenenfalls mit den Einschränkungen, die für die Behandlung von somatisch kranken Personen getroffen werden müssen [160].

Besonders ist darauf hinzuweisen, dass für die meisten Beschwerden im Kontext von Long COVID eine Tendenz zur Besserung im Laufe der Zeit festzustellen ist [109] und dass bei leicht ausgeprägten depressiven Symptomen eine medikamentöse Behandlung nur unter bestimmten Umständen empfohlen wird [161]. Es wird sich daher in allen Fällen mit nicht stark ausgeprägter Symptomatik eine zuwartend-beobachtende Vorgangsweise empfehlen. Oft sind Angst und Depression Folge aktueller sozialer Probleme und brauchen eine einen Lösungsansatz auf dieser Ebene [113]. Für PTSB ist eine spezifische Psychotherapie (z. B. eye movement desensitization and reprocessing – EMDR) die Behandlung erster Wahl.

#### 12.4.10. Weitere Behandlungsansätze

Es werden eine Reihe von Behandlungsansätzen angeboten, wie Nahrungsergänzungsmittel, pflanzliche Wirkstoffe, homöopathische Mittel, wo Belege für eine Wirksamkeit fehlen. Auch bei diesen Substanzen können schädliche Neben- und Wechselwirkungen nicht grundsätzlich ausgeschlossen werden [9].

##### Empfehlung

Wenn von Patient:innen Wünsche nach nicht überprüften therapeutischen Konzepten geäußert werden, sollten diese auf mögliche schädliche Wirkungen überprüft werden (soweit dies möglich ist), und ansonsten offen und realistisch erklärt werden, dass es keine Belege für deren Wirksamkeit gibt, und darauf aufmerksam gemacht werden, wenn sich mögliche schädliche Wirkungen nicht ausschließen lassen.

## 13. Nachsorge und Rehabilitation

### 13.1. Allgemeines

Der übliche Weg nach COVID‑19 ist die Wiederaufnahme der Berufs- bzw. Alltagstätigkeit nach Ende der Isolierung (je nach Situation 10–14 Tage). Eine Gesundschreibung bei Berufstätigen ist dafür, anders als bei anderen Erkrankungen, nicht notwendig, da aufgrund des behördlichen Bescheides die Ausstellung einer Krankschreibung nicht vorgesehen ist.

Bekannt ist jedoch, dass eine relevante Anzahl der Betroffenen auch nach als mild bezeichneten Verläufen ohne Hospitalisierungsnotwendigkeit zu diesem Zeitpunkt noch an Symptomen leidet, in einer österreichischen Studie waren es 70 % – davon über 40 %, die mehr als ein Symptom angaben (Sample aus der Primärversorgung), incl. 9 % hospitalisierte Patient:innen [148], eine grundsätzliche Abklärung vor Wiederaufnahme der beruflichen Tätigkeit sollte daher erwogen werden.

Die Differenzierung **organspezifischer Langzeitfolgen** (irreversibel oder Verschlechterung vorbestehender chronischer Erkrankungen) von funktionellen, meist reversiblen Folgezuständen ohne strukturelle Erkrankung ist für die Entscheidung zur Rückkehr zu körperlich beanspruchenden Aktivitäten (Beruf, Sport oder andere Aktivitäten) wesentlich, s. dazu Kap. 8. Strukturelle Schädigungen z. B. von Herz oder Lunge müssen abgeklärt und behandelt werden, die Belastungsgrenzen müssen unter medizinischer Begleitung oder im Rahmen einer Rehabilitation ausgelotet werden.

**Funktionelle Störungen** benötigen meist Geduld und allgemein-rehabilitative Maßnahmen. Rückkehr- und Trainingspläne werden entsprechend Befunden und Klinik ausgerichtet, die Schwere des Verlaufs der Akuterkrankung spielt demgegenüber eine untergeordnete Rolle [8, 58, 162, 163].

Wesentlich für weitere Entscheidungen ist die Beurteilung der Beeinträchtigung von Alltags- und Arbeitsfähigkeit aufgrund der Symptomatik und der subjektive Leidensdruck. Anmerkung: die Symptomatik kann stark fluktuieren [8]. Die Post-COVID‑19-Skala des funktionellen Status ist ein geeignetes Tool (Abb. 4; [137]).

#### Empfehlung

Die Beratung hinsichtlich Selbstmanagements und die Planung des „Weges zurück“ in Alltag, Sport und Arbeit erstreckt sich auf folgende zentrale Aspekte:

- Festlegung realistischer Ziele
- Klare Vereinbarungen über Belastungsgrenzen/vorzeitigen Kontrollen
- Strukturierte hausärztliche Betreuung und Behandlungsplanung gemeinsam mit der Patient:in und/oder deren Angehörigen bzw. Betreuungspersonen.
- Es bedarf Ruhe und Zeit, ein überhastetes „Zuviel wollen“ bringt keinen Benefit, sondern erhöht das Risiko für längere chronische Verläufe und/oder akute Exazerbationen.

### 13.2. Wiedereingliederung im häuslichen Setting

#### 13.2.1. Rückkehr in den Alltag (ohne wesentliche körperliche Belastung)

- Alle Patient:innen nach COVID‑19 sollten darüber aufgeklärt sein, dass persistierende Symptome auch nach mildem und moderatem Verlauf möglich sind, dass diese sich aber in den allermeisten Fällen im Verlauf von einigen Wochen, längstens Monaten zurückbilden.
- Einschränkungen der Leistungsfähigkeit sollten besprochen bzw. je nach Ausmaß abgeklärt werden (s. Abschn. 10.1. und 10.2.). Dies liegt in der Verantwortung der hausärztlichen Primärversorgung.
- Die Kernpunkte (nach Ausschluss relevanter struktureller Folgeschäden) sind:
  - Ist die Bewältigung der täglichen Aktivitäten möglich? [162]
  - Wie hoch ist die Alltagsbelastung (Gemeinsame Abschätzung): Ausmaß der körperlich erforderlichen Fitness? Störungen der Kognition relevant für Freizeitbeschäftigungen/Bedienen von Maschinen oder Transportmitteln?

Als Technik zur Wiedererlangung von Alltagsfähigkeiten kann das „Pacing“ eingesetzt werden (s. Kap. 12):

- Belastungsbeginn: Spazieren (langsame Steigerung von Spazierdauer und Tempo etc.), langsame Steigerung der alltäglichen Belastung (vom Kochen zum Einkaufen, vom Zusammenräumen zum Putzen)
- Bei Verschlechterung der Symptome: Pause und Rückkehr zum absolvierbaren Niveau nach Abklingen der akuten Beschwerdesymptomatik („Symptomtitriertes Training“)
- Dies gilt analog für kognitive Leistungen und mentale und emotionale Belastungen
- Physio- Ergo-, und/oder klinisch-psychologische Unterstützung kann nach Bedarf angeboten werden.
- Persönliche Leistungs- bzw. Belastungsgrenzen müssen grundsätzlich respektiert werden.
- Zu beachten ist auch, dass Reizüberflutung vermieden werden sollte (Pausen, Schlafhygiene, „Bildschirmhygiene“ etc.)

#### 13.2.2. Wiederaufnahme des Sports

Auch für die Freigabe zum Sport muss, das individuelle Risiko bewertet werden.

Liegen nach Ablauf der Quarantäne noch Symptome vor, erfolgt die Abklärung entlang der im Kap. 9 Differenzialdiagnostik aufgeführten Schritte. Vor allem wenn eine kardiale Beteiligung vermutet wird, muss eine Abklärung unbedingt erfolgen.

Bei Symptomfreiheit nach der akuten Phase der Erkrankung gilt ansonsten:

##### Amateursport

Selbst bei vollständiger Genesung sollte die Rückkehr zum Sport **frühestens 7 Tage** nach Erlangen der Symptomfreiheit angestrebt werden und über 2 Wochen mit minimaler Belastung stattfinden [162], die weitere Belastungssteigerung sollte auch in ausreichend großen Intervallen (zumindest wöchentlich) erfolgen. Bevor eine Rückkehr zur sportlichen Belastung angedacht werden kann, sollten die Aktivitäten des täglichen Lebens und die Absolvierung einer Wegstrecke von 500 m in der Ebene ohne Erschöpfungszeichen oder Atemnot absolviert werden können [164].

Mit zunehmender Schwere der Erkrankung steigt auch die Wahrscheinlichkeit für etwaige Komplikationen/Spätfolgen. Im Konsensuspapier der sportmedizinischen Universitäts- und Landesinstitute Wien, Salzburg und Innsbruck wird daher relativ pragmatisch unterschieden[165].

Als einfache Variante des Selbstmonitoring bei Belastung wird in der Literatur die Borg-RPE-Skala oder Borg-CR10-Skala (Tab. 1: Borgskala) für Laien angegeben. Weitere Hilfestellung können Ruhepuls und Pulsraten sein. Wichtig sind klare Vereinbarungen, wann sofort medizinischer Rat gesucht werden sollte. Kommt es zum Auftreten von Symptomen, sollte eine Belastungspause bis zu einer Symptomfreiheit von 24 h gemacht werden und auf die vorherige Belastungsstufe – welche ohne Symptome bewältigt wurde – zurückgegangen werden. Nähere Details und hilfreiche Tipps dazu: BMJ – Return to play [164]und ins Deutsche Deutsche übersetzt COVID‑19 und return to play – Sportärztezeitung [166].

##### Empfehlung

Die Rückkehr zum Sport sollte auch bei völliger Genesung frühestens 7 Tage nach Erlangen der Symptomfreiheit angestrebt werden und über 2 Wochen mit minimaler Belastung stattfinden. Die weitere Belastungssteigerung sollte auch in ausreichend großen Intervallen (zumindest wöchentlich) erfolgen. Je schwerer die Akuterkrankung verlief, desto vorsichtiger ist der Weg zurück zu planen.

##### Leistungssport

Für Athleten wurde die Wiederaufnahme des Sports nach Infektionskrankheiten untersucht, hier gibt es Leitlinien aus dem Jahr 2019 der Europäischen Kardiologischen Gesellschaft [167], die nach Infekt ein

- 12 Kanal EKG
- Echo
- BB, Troponin, CRP

empfehlen. Wenn diese negativ sind, dann gilt das Risiko eines kardiovaskulären Events in der Zukunft als sehr gering. In speziellen Fällen (Myokarditissymptome, Auffälligkeiten im Echo) sollte eine MRT des Herzens angeschlossen werden [167, 168]. Von pneumologischer Seite ist eine symptomorientierte Untersuchung (s. Abschn. 10.4.) ausreichend.

Evidenz speziell hinsichtlich der Situation bei Long COVID fehlt noch. In jedem Zweifelsfall und nach schweren Verläufen sollte eine spezialisierte sportmedizinische Begutachtung erfolgen.

#### 13.2.3. Rückkehr an den Arbeitsplatz

- Für körperlich stark beanspruchende Tätigkeiten gelten sinngemäß die Empfehlung wie für die Wiederaufnahme des Sports.
- Nicht zu vergessen: Auch für die Modalität des Arbeitsweges (Gehen, Fahrrad fahren u. ä.) gelten die gleichen Kriterien wie für den Beginn der sportlichen Belastung.
- Belastungsgrenzen und Berufseignung bei anhaltenden starken Einschränkungen sollten während einer Rehabilitation erhoben werden, und je nach Situation vor Arbeitsantritt mit den zuständigen Präventivkräften im Betrieb (Sicherheitsfachkraft, Betriebsärzt:in) und den zuständigen Institutionen (Arbeitsinspektion, AUVA) besprochen werden.
- In vielen Fällen können (vorübergehende) Anpassung von Arbeitsplatz und Arbeitsbedingungen den Wiedereintritt ins Berufsleben erleichtern bzw. vorverlegen. Auch hier sind die Präventivkräfte am Zug, idealerweise in Kooperation mit den hausärztlichen Primärversorger:innen
- Eine Krankschreibung erfolgt nach den gleichen Grundsätzen wie immer, das Kriterium ist die tatsächliche, anforderungsbezogene Leistungsfähigkeit der Betroffenen. Die Diagnose sollte sich auf das jeweilige dominierende Symptom beziehen, da „Long COVID“ derzeit noch als Komplex äußerst unterschiedlicher Symptome zu sehen ist, und als klare Diagnose noch nicht ausreichend definiert.

Eine Schwierigkeit, die sich aufgrund der derzeitigen Überlastung der Rehabilitationsstrukturen ergibt, ist die Überbrückung der Zeitspanne bis zum Antritt der Rehabilitation. Eine Arbeitsaufnahme in dieser Zeit wird für körperlich arbeitende Personen meist nicht gefahrlos möglich sein, woraus sich Probleme ergeben können. Die Begutachtung durch und gemeinsame Entscheidung mit Spezialisten je nach Gegebenheiten ist dringend empfohlen. Auch die Kontaktnahme mit Arbeitnehmerschutzeinrichtungen (AK, Gewerkschaft) sollten den Betroffenen angeraten werden. Case Manager der Krankenkassen können bei der Organisation der Wiedereingliederung unterstützen, soweit solche verfügbar sind.

Spekulative Annahmen über eine tatsächliche Arbeitsrückkehr sollten gegenüber den Arbeitgebenden aufgrund der unklaren Krankheitsdauer bei Long COVID vermieden werden (daher max. „voraussichtliche Rückkehr“), es sollte eher möglichst konstruktiv über eine gestufte Rückkehr in den Arbeitsprozess mit dem Arbeitgebenden gesprochen werden (Rechtsgrundlagen: https://www.arbeiterkammer.at/krankenstand).

Bei Inanspruchnahme von Wiedereinstiegsmodellen wie die Wiedereingliederungsteilzeit (ÖGK) oder den Diensterleichterungen (BVAEB) empfiehlt es sich daher, von Beginn an den maximal möglichen Zeitraum anzunehmen: Verkürzt kann bei schnellerer Genesung immer werden, ein Verlängern des schrittweisen Wiedereinstiegszeitraums ist nur schwer möglich.

##### Empfehlung

Für die Planung der Rückkehr an den Arbeitsplatz sind neben Schwere der Akuterkrankung und weiterbestehender Symptomatik auch die individuellen Arbeitsplatzanforderungen und Arbeitsbedingungen zu berücksichtigen.

### 13.3. Rehabilitation

#### 13.3.1. Indikation

Anhand der Post-COVID‑19-Skala des funktionellen Status (Abb. 4) lässt sich rasch die Indikation für rehabilitative Maßnahmen festmachen. Ab dem Stadium 2 können nach der ärztlichen Abklärung je nach Schweregrad Rehabilitationsverfahren beantragt werden, um eingeschränkte Körperfunktionen, und Aktivitäten zu verbessern und eine bestmögliche Teilhabe in sozialer und beruflicher Hinsicht zu erreichen.

Besteht Rehabilitationsbedarf aufgrund pneumologischer, neurologischer, psychiatrischer oder kardiologischer postinfektiöser Schädigungen, kann eine indikationsspezifische Rehabilitation erfolgen. Die Therapien werden je nach Einschränkung von Körperfunktionen und Aktivitäten geplant und fokussieren auf die bestmögliche Teilhabe. Rehabilitationsverfahren erfolgen multimodal unter Einbeziehung verschiedener Fachdisziplinen.

Hierzu zählen z. B.: Physiotherapie, Trainingstherapie, Ergotherapie, Psychologie, Logopädie, Diätologie, Massage.

Die WHO teilt die Rehabilitation in 4 Phasen.

- **Phase I** entspricht der Mobilisation im Krankenhaus.
- Die **Phase II** kann als Anschlussheilverfahren entweder ambulant oder stationär erfolgen.
- Die **Phase III** ist eine Anschlussrehabilitation, die ambulant erfolgt, um die Nachhaltigkeit der Phase II Rehabilitation zu verbessern und die Situation bei schwereren Verläufen zu stabilisieren.
- Die **Phase IV** bedeutet eine „Verstetigung“ in dem Sinne, als die Patientin/der Patient das Erlernte ein Leben lang weiterführen sollte.

#### 13.3.2. Evidenz

##### Pneumologisch

Long COVID mit **Dyspnoe, körperlicher Minderbelastbarkeit und/oder Fatigue** kann sowohl bei Patient:innen nach einem kritischem, aber auch nach einem milden Verlauf bestehen bleiben. Erste Publikationen konnten die Machbarkeit, Sicherheit und Effektivität von Rehabilitationsmaßnahmennach einem schweren Verlauf zeigen [169–171]. Die Leistungsfähigkeit sowie lungenfunktionelle Einschränkungen konnten verbessert werden.

Nach einer stationären 3‑wöchigen pneumologischen Rehabilitation verbesserten sich sowohl körperliche Leistungsfähigkeit klinisch relevant (6-Minuten Gehtest): mittelschwer Betroffene +48 m [95 % Konfidenzintervall, KI 35–113 m], schwer Betroffene +124 m [75–145 m] [169] um im Mittel ca. 100 m, wie auch psychischen Parameter wie Angst, Depression und Flash-backs. Dieselben Erfahrungen haben wir in 6 Wochen ambulanter Rehabilitation gemacht (Verbesserung 6 Minutengehtest – NNT 1.4, Reduktion Fatigue – NNT 1.9, Verbesserung Dyspnoe – NNT 1.8, Verbesserung der PCFS – NNT 1.2; in press). Weitere Studien zeigten eine Verbesserung der restriktiven Lungenfunktionsveränderungen, der Diffusionsstörung und der Atemmuskelkraft.

##### Neurologisch

Long COVID Patient:innen **mit Störungen von globalen oder spezifischen mentalen Funktionen, der Sprache, des Schluckens, der Motorik oder Sensorik **sollten einer neurologischen Evaluation und/oder neurorehabilitativen Versorgung zugeführt werden.

Bei kritischen Verläufen stellt das Post-Intensive-Care-Syndrom (PICS) eine bekannte und häufige Folge dar, die Einschränkungen auf die gesundheitsbezogene Lebensqualität und Teilhabe zu Folge hat [172]. Diese Patient:innen bedürfen nach klarer Definition von Rehabilitationszielen einer Früh‑/Rehabilitation.

**Kognitive Störungen** beim PICS [173] ebenso wie nach mildem oder moderatem Verlauf betreffen gehäuft Aufmerksamkeits- und Gedächtnis- sowie Exekutivfunktionen [174].

Zudem können in Zusammenhang mit COVID‑19 verschiedene weitere spezifische Erkrankungen wie Schlaganfälle, Enzephalomyelitiden, ein Guillain-Barré-Syndrom (GBS), ein Miller Fisher-Syndrom, Hirnnerven-Neuritiden, Myositiden, eine Myasthenia gravis und Plexopathien auftreten, die alle mit spezifischem Rehabilitationsbedarf einhergehen.

##### Kardiologisch

COVID‑19 kann mit schwerwiegenden kardiovaskulären Erkrankungen wie einer Myokarditis, einer Herzinsuffizienz, einem akuten Koronarsyndrom (ACS), Arrhythmien oder venösen Thromboembolien einhergehen [175, 176]. In diesen Fällen kann eine kardiologische Rehabilitation eingeleitet werden. Die Inhalte der kardiologischen Rehabilitation richten sich nach den Hauptindikationen wie Herzinsuffizienz, ACS, Myokarditis und thromboembolischen Erkrankungen (siehe S3-Leitlinie zur kardiologischen Rehabilitation 2020 [177]).

Nach Ausloten der individuellen Belastungsgrenzen wird in Abhängigkeit von der zugrundeliegenden kardiologischen Problematik ein multimodales individuell adaptiertes Training bestehend aus Ausdauertraining (am Fahrradergometer und/oder als Gehtraining) sowie ein Krafttraining jeweils mehrmals pro Woche angeboten. Bei schwer Betroffenen nach protrahierten Intensivaufenthalten kann auch ein Training der Inspirationsmuskulatur zur Anwendung kommen. Im Vordergrund steht die Erhöhung der Belastungstoleranz zur Förderung der bestmöglichen Teilhabe. Zusätzlich erfolgt eine diätologische sowie häufig auch eine psychologische Betreuung.

##### Psychiatrisch

Eine multimodale psychiatrische Behandlung ist angezeigt bei klinisch relevanten psychischen Krankheiten im Rahmen von Long-COVID wie Anpassungsstörung, Depression, Angststörung, Somatisierungsstörung, Zwangsstörung, Psychose oder PTSD. Diese umfasst unter anderem Psychopharmaka, Psychotherapie, Physiotherapie, Sozialarbeit und Arbeit mit dem sozialen Umfeld. Wenn ambulante Rehabilitationsmaßnahmen nicht ausreichen, ist eine stationäre Rehabilitation indiziert.
